# Supplementary material for: Creating solvation environments in heterogeneous catalysts for efficient biomass conversion
Source: Nat Commun. 2018 Aug 13;9:3236. doi: 10.1038/s41467-018-05534-5 (PMC6089952; doi:10.1038/s41467-018-05534-5)
Supplement: Supplementary file 1 — Supplementary Information [file 41467_2018_5534_MOESM1_ESM.pdf]

# Supplementary Information

## Creating Solvation Environments in Heterogeneous Catalysts for Efficient Biomass Conversion

Sun et al.

## Supplementary Figures

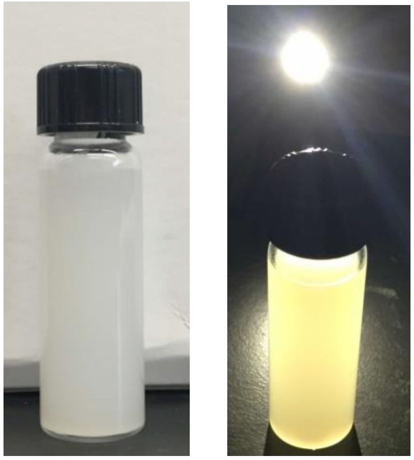

**Supplementary Figure 1 | Digital images of PSS-IL dispersed in THF.** (left) under natural lighting and (right) under flashlight.

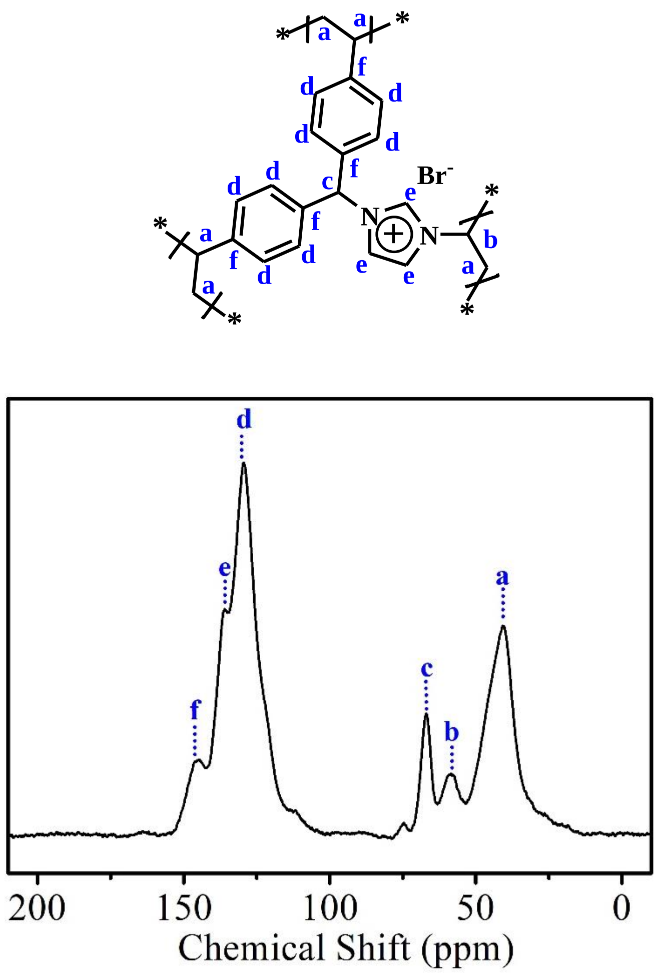


**Supplementary Figure 2 | $^{13}C$ MAS NMR spectrum of PSS-IL.**

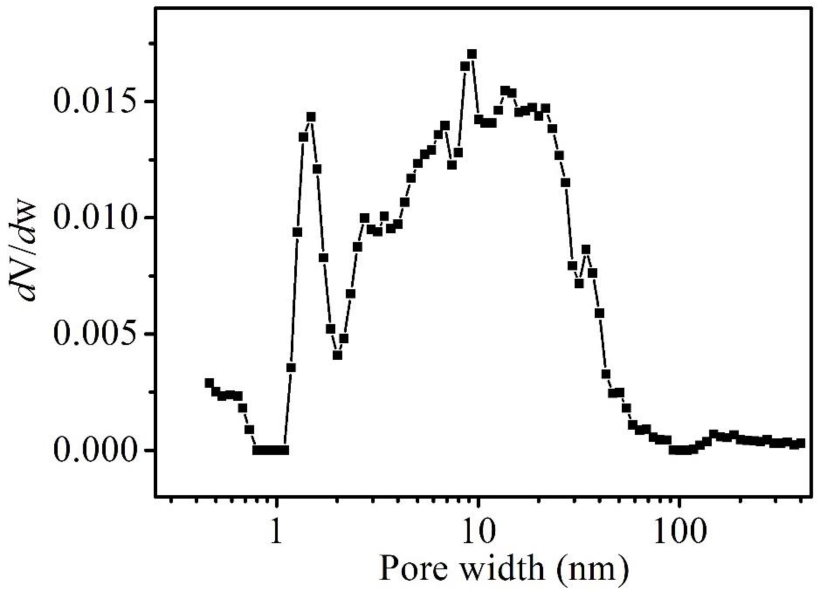


**Supplementary Figure 3 | Pore size distribution of PSS-IL**. Calculated by the nonlocal density functional theory method (NLDFT).

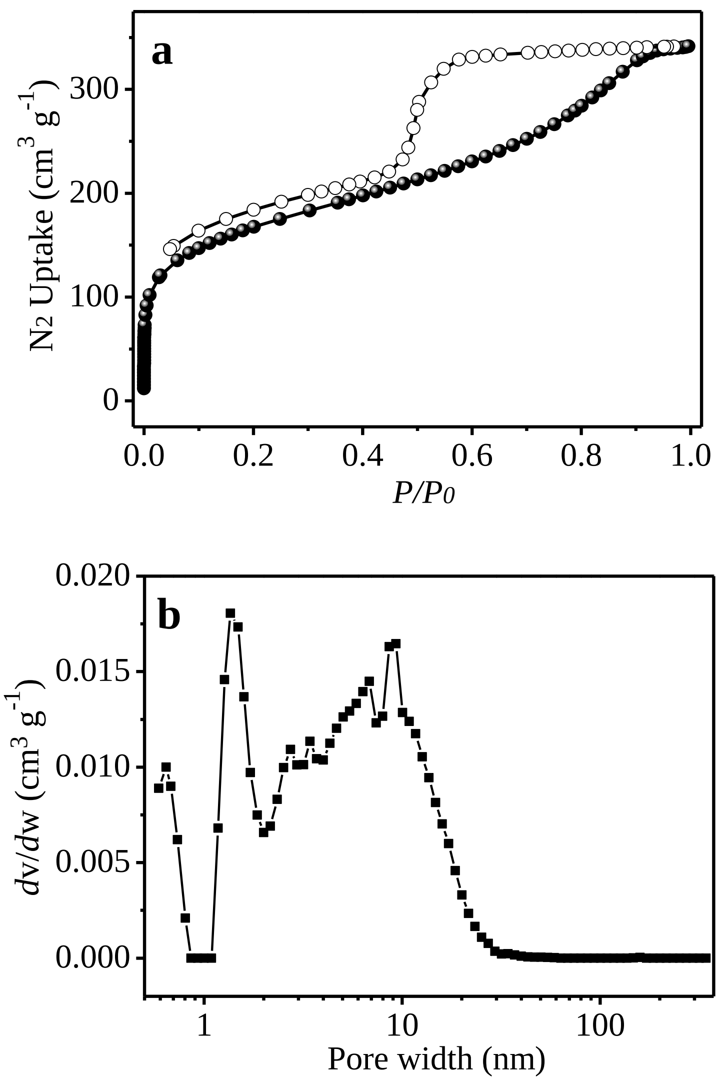


**Supplementary Figure 4 | Textural parameters.** (a) $N_2$ sorption isotherms and (b) pore size distribution of PSS-NMP calculated based upon the nonlocal density functional theory method (NLDFT).

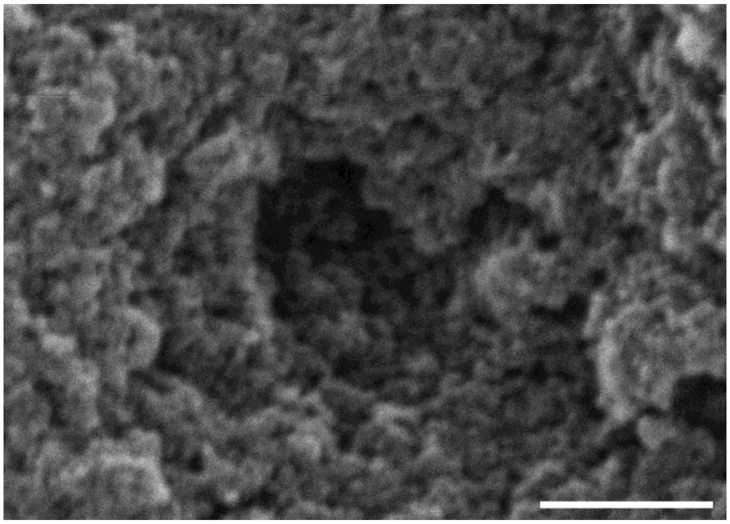

**Supplementary Figure 5 | SEM image of PSS-NMP (scale bar denotes 500 nm).**

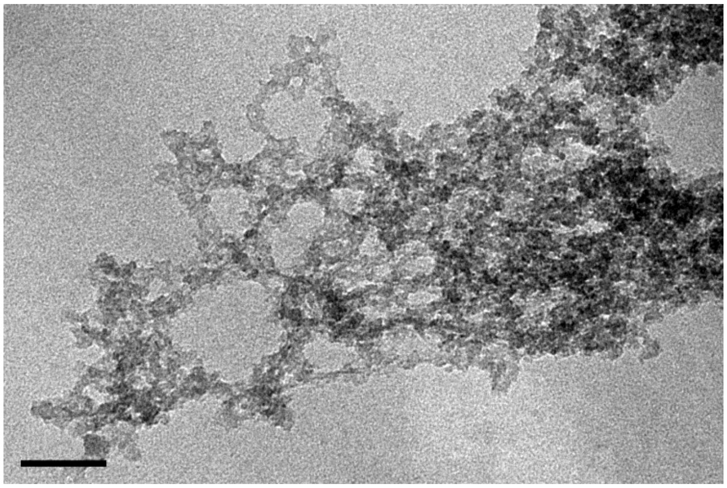

**Supplementary Figure 6 | TEM image of PSS-NMP (scale bar denotes 100 nm).**

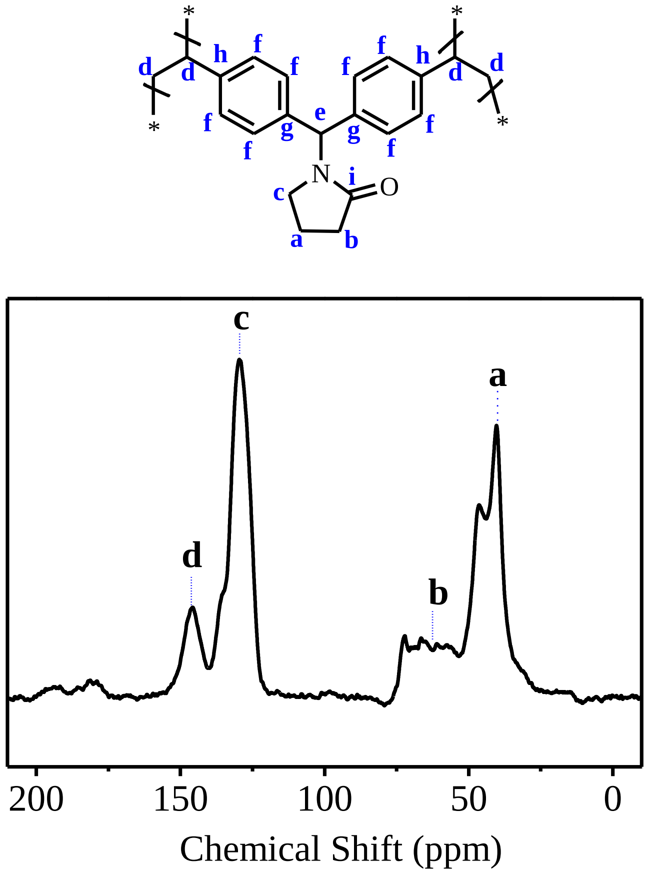


**Supplementary Figure 7 | $^{13}C$ MAS NMR spectrum of PSS-NMP.**

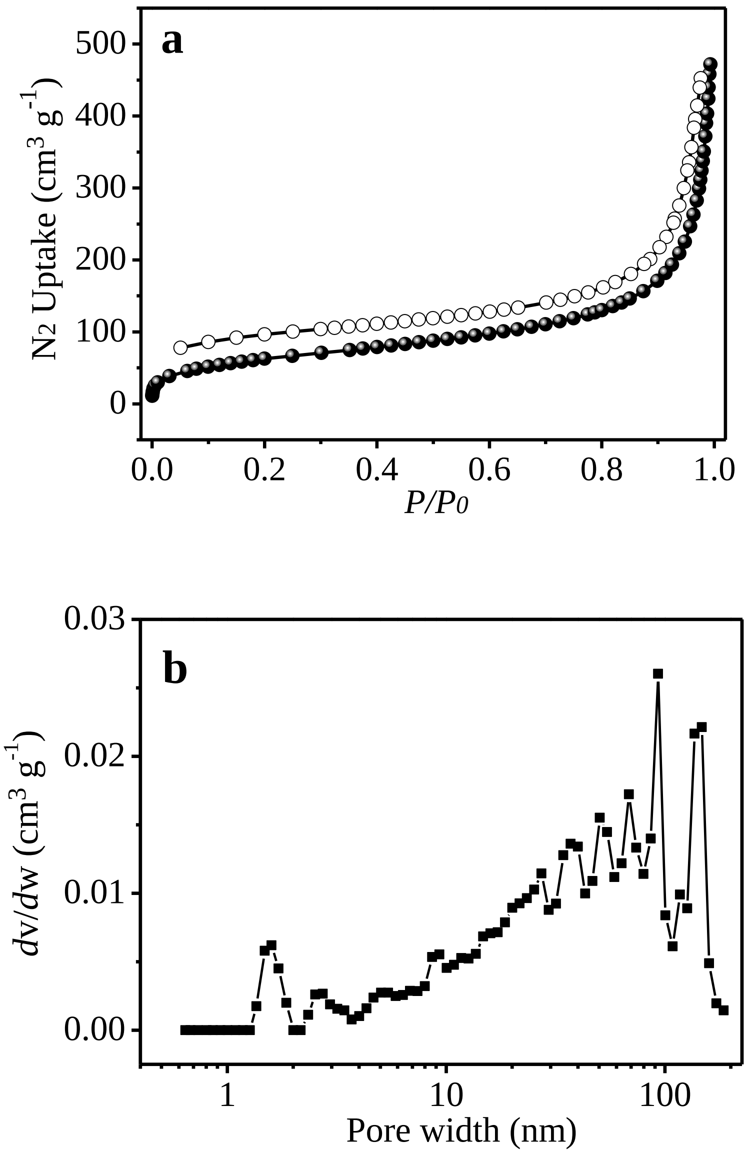


**Supplementary Figure 8 | Textural parameters.** (a) $N_2$ sorption isotherms collected at -196 °C and (b) pore size distribution of PSS-DMSO calculated by the nonlocal density functional theory method (NLDFT).

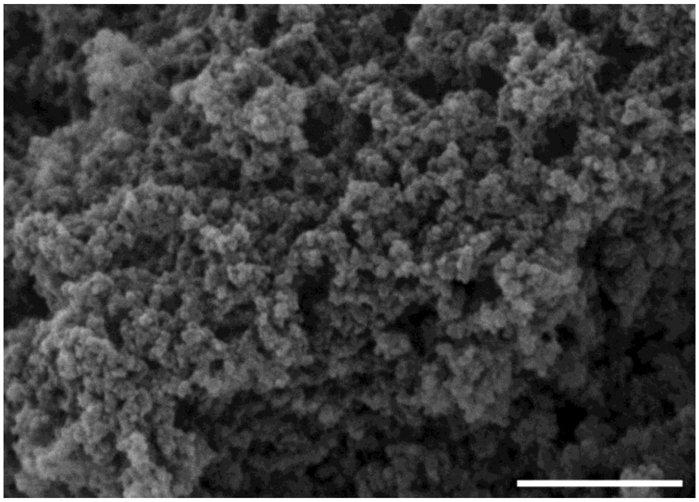

**Supplementary Figure 9 | SEM image of PSS-DMSO (scale bar denotes 500 nm)**

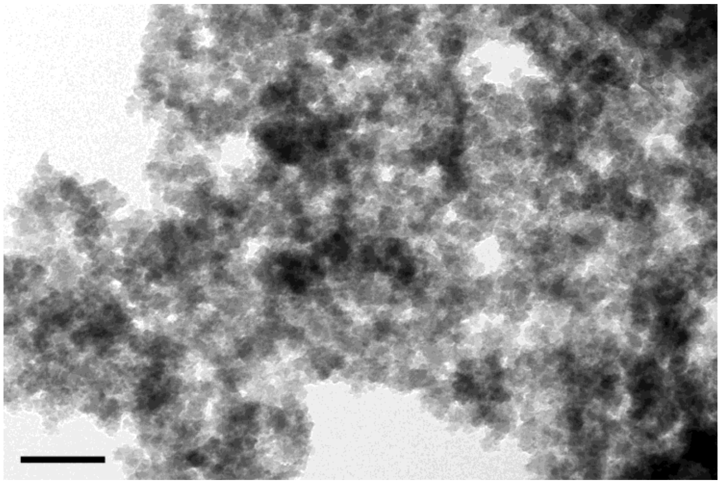

**Supplementary Figure 10 | TEM image of PSS-DMSO (scale bar denotes 200 nm).**

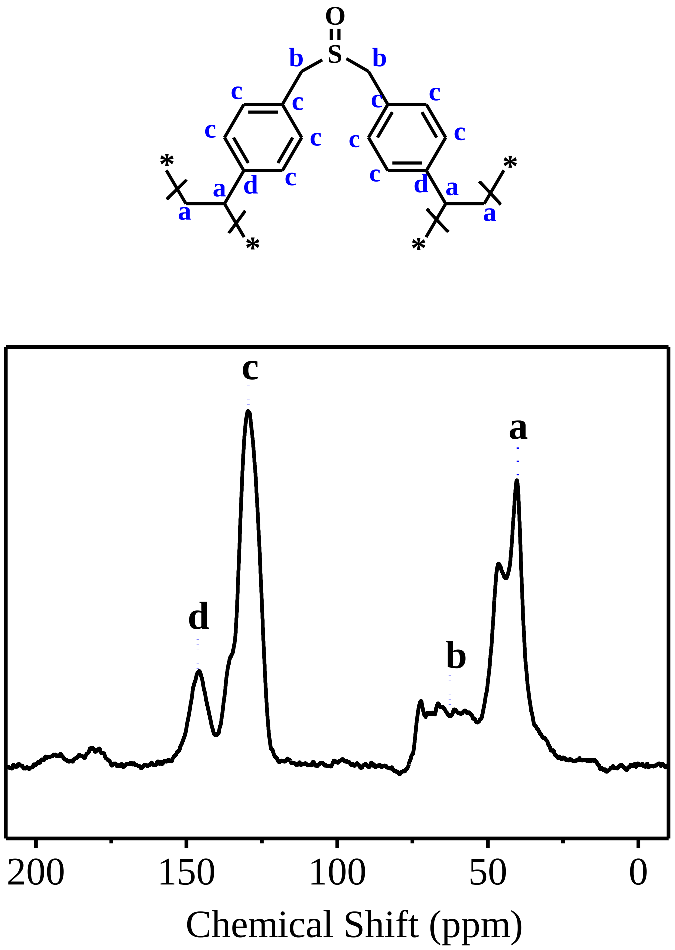


**Supplementary Figure 11 | $^{13}$C MAS NMR spectrum of PSS-DMSO.**

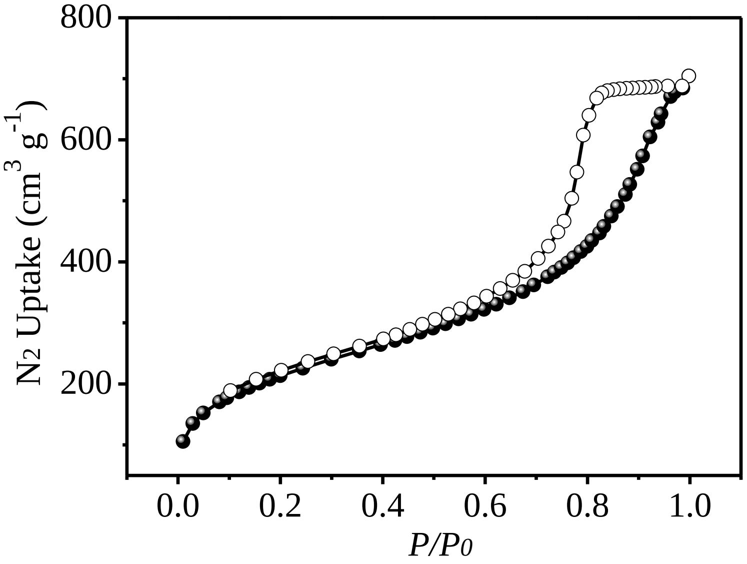


**Supplementary Figure 12 | $N_2$ sorption isotherms of polydivinylbenzene (PDVB) collected at -196 °C.** The BET surface area and pore volume were calculated to be 743 $m^2$ $g^{-1}$ and 1.09 $cm^3$ $g^{-1}$, respectively.

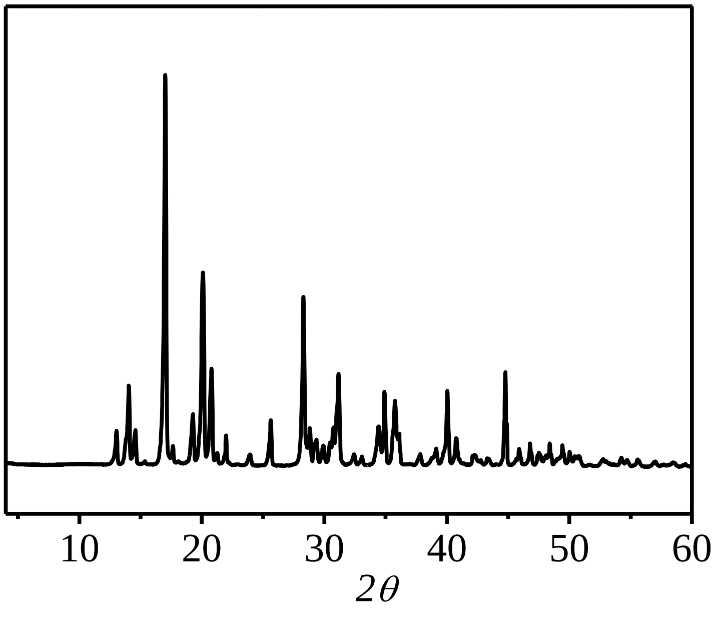


**Supplementary Figure 13 | XRD pattern of fructose.**

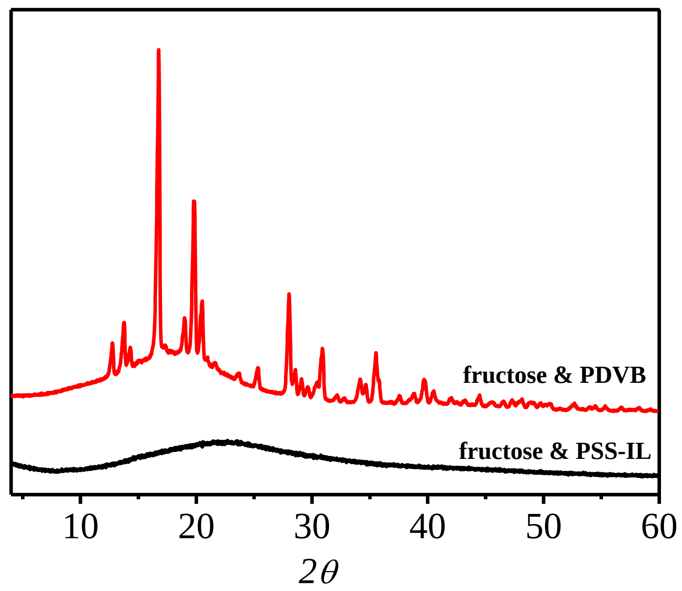


**Supplementary Figure 14 | XRD patterns of finely grinded PDVB or PSS-IL (100 mg) and fructose (10 mg).**

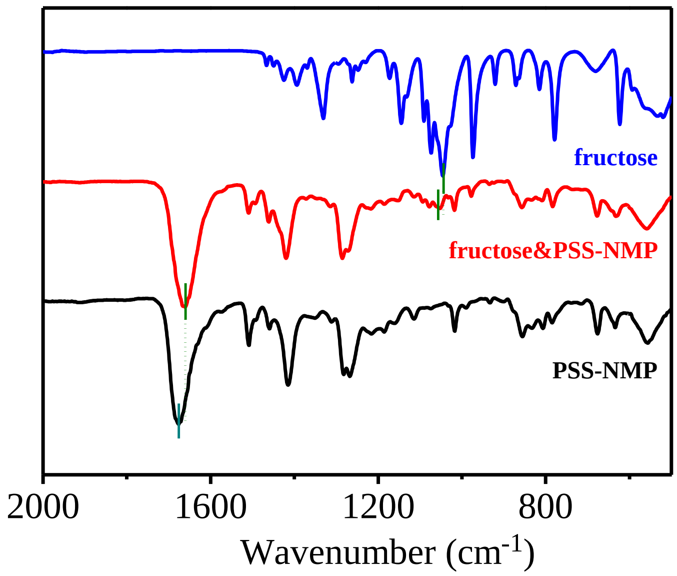


**Supplementary Figure 15 | IR spectra of PSS-NMP, composite of finely grinded fructose and PSS-NMP (fructose&PSS-NMP), and fructose.**

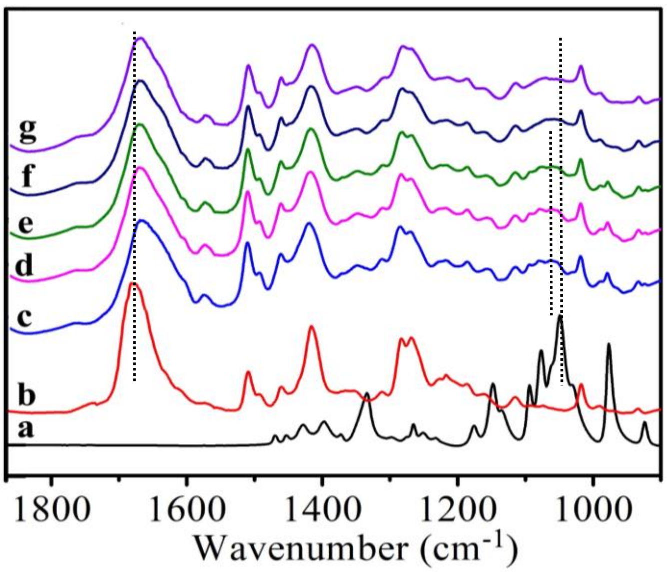


**Supplementary Figure 16 | IR spectra of (a) fructose, (b) PSS-NMP, and variable temperature IR spectra of (c) RT, (d) 50 ºC, (e) 100 ºC, (f) 150 ºC, and (g) 200 ºC of the composite of finely grinded fructose (10 mg) and PSS-NMP (100 mg).** Negligible changes in the IR spectra of fructose@PSS-NMP were observed even after heating up to 100 °C, suggestive of the strong binding. Further increasing the temperature to 150 °C, changes were observed in the range of 1000 $cm^{-1}$ to 1100 $cm^{-1}$, likely due to fructose melting at this temperature.

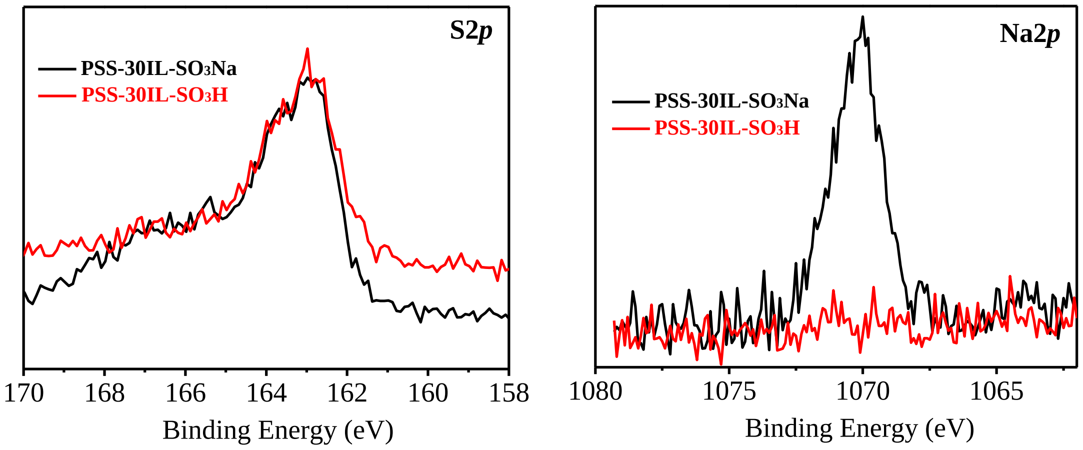


**Supplementary Figure 17 | XPS spectra.** The presence of sulfur and sodium signals suggests the successful incorporation of sodium sulfonate species. Negligible sodium species in PSS-30IL-$SO_3$H samples confirm the complete cation-exchange process.

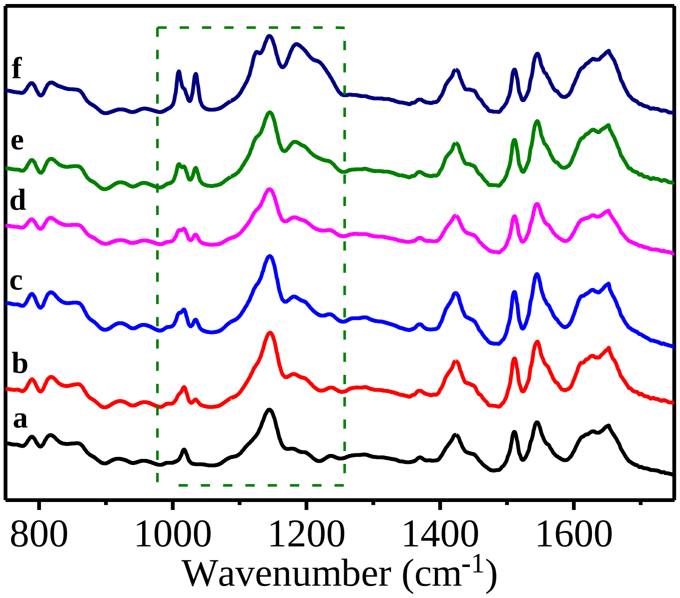


**Supplementary Figure 18 | IR spectra of PSS-xIL-$SO_3H$ with different mole ratios of ionic moieties to sodium *p*-styrene sulfonate (x).** (a) ∝, (b) 30, (c) 20, (d) 15, (e) 10, and (e) 5, respectively. The intensities of bands associated with S=O and C-S increase along with the mole ratio of ionic moiety to $SO_3H$ group.

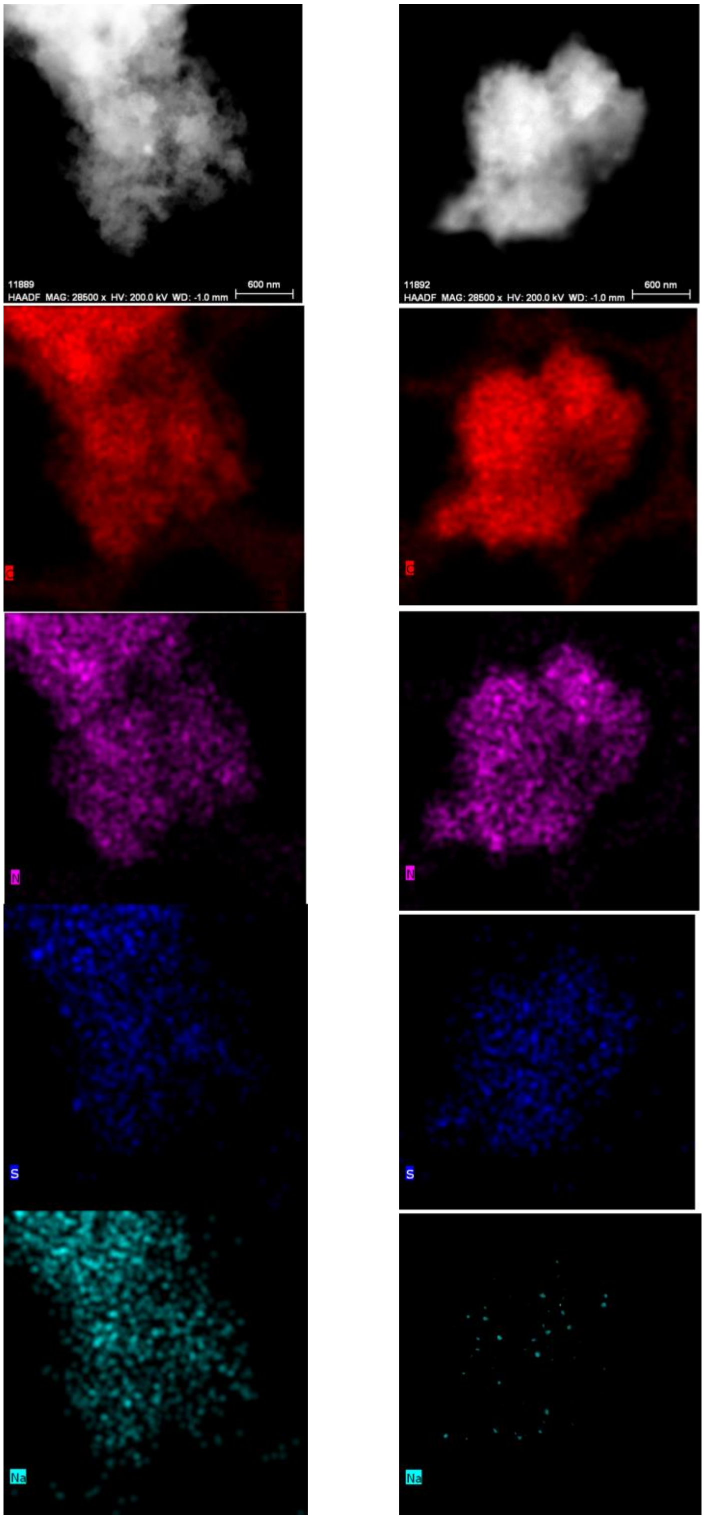


**Supplementary Figure 19 | HADDF-STEM and corresponding element mapping of PSS-30IL-$SO_3Na$ (left) and PSS-30IL-$SO_3H$ (right).**

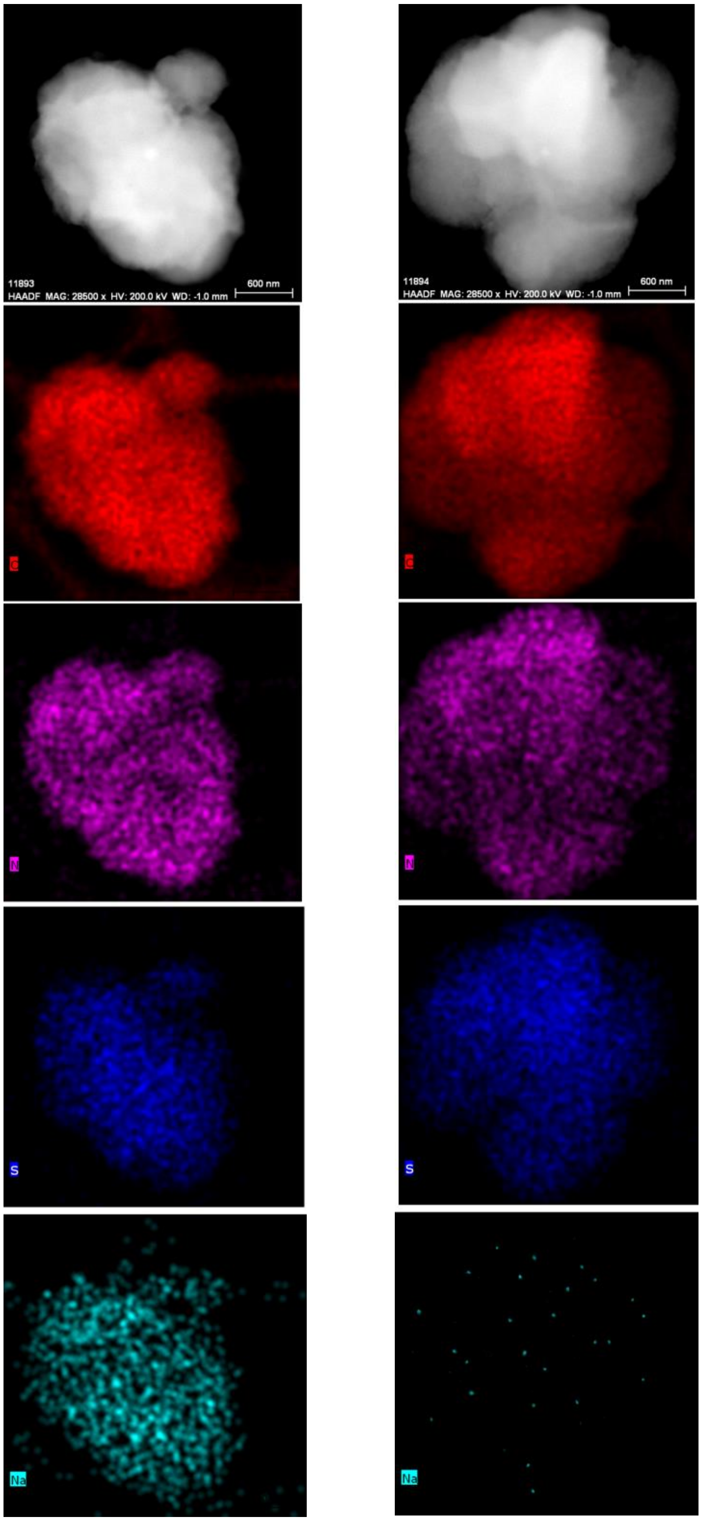


**Supplementary Figure 20 | HADDF-STEM and corresponding element mapping of PSS-5IL-$SO_3$Na (left) and PSS-5IL-$SO_3$H (right).**

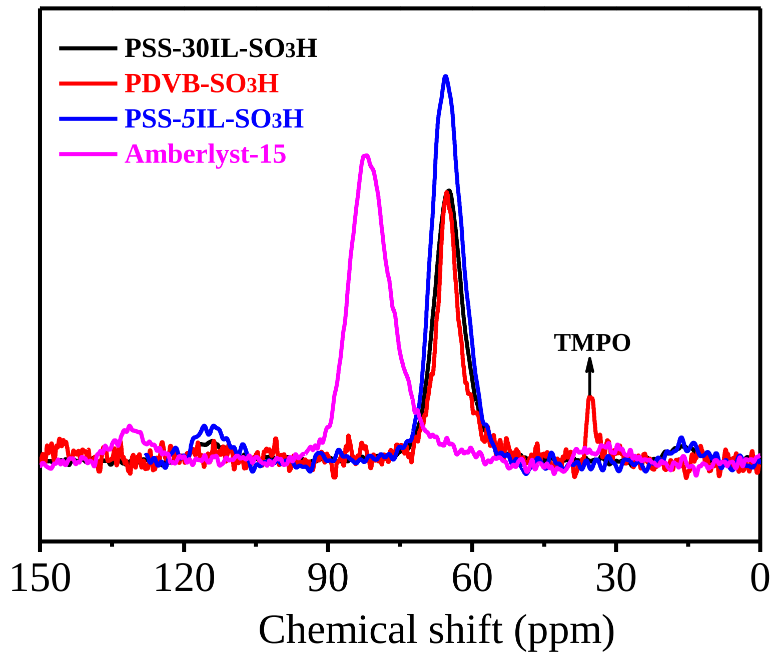


**Supplementary Figure 21 | $^{31}P$ MAS NMR spectra of TMPO chemically adsorbed on various samples.** $^{31}P$ MAS NMR spectra of TMPO after interaction with PSS-30IL-$SO_3H$ and PSS-5IL-$SO_3H$ samples show a singlet peak at 64.8 and 65.3 ppm, respectively, appearing to possess a moderate strength, which are much weaker than that in Amberlyst-15. The narrow NMR signal suggests the homogeneity of the acid sites in these samples.

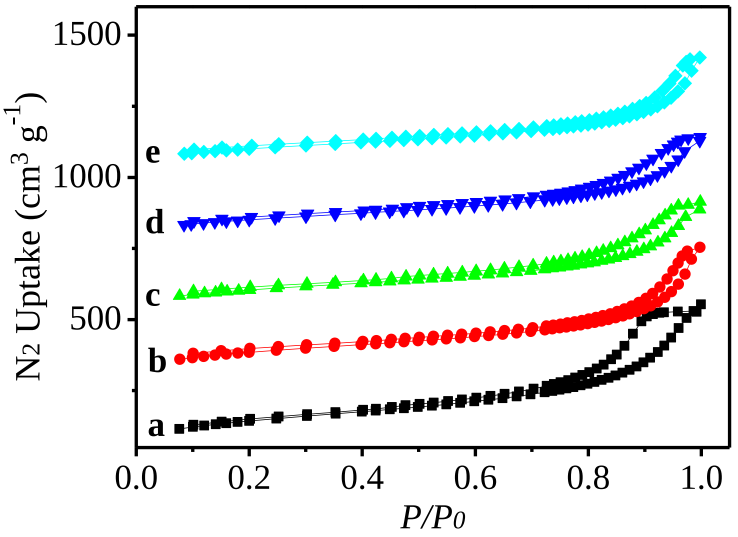


**Supplementary Figure 22 | $N_2$ sorption isotherms of the PSS-xIL-$SO_3H$ with different mole ratios of ionic moieties to sodium *p*-styrene sulfonate (x).** (a) 30, (b) 20, (c) 15, (d) 10, and (e) 5, respectively. Isotherms of (b)-(e) have been offset by 250, 500, 750 and 1000 $cm^3\ g^{-1}$, respectively, along the vertical axis for clarity.

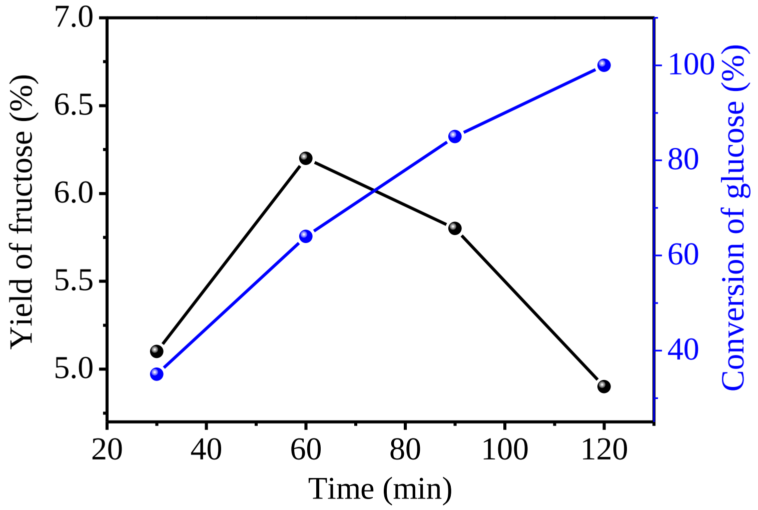


**Supplementary Figure 23 | The profile of HMF yield as a function of reaction time over PSS-30IL-$SO_3$H in the absence of solvent.** Reaction conditions: PSS-30IL-$SO_3$H (100 mg), fructose (100 mg), and 120 °C. The figure above shows that HMF yield is always below 7% within 2 h, at which time fructose reached full conversion. It is noteworthy that the color of the reaction mixture turned from light yellow to dark brown, indicative of the occurrence of side reactions, probably due to the instability of HMF under solvent-free and heating conditions, which also has been noted in the reference (*Angew. Chem. Int. Ed.* 2016, *55*, 8838); while, in the presence of THF, the yield of HMF can reach to 98.8%, indicating that solvent is still needed for mass transfer to bring the reaction under control, at least in this case.

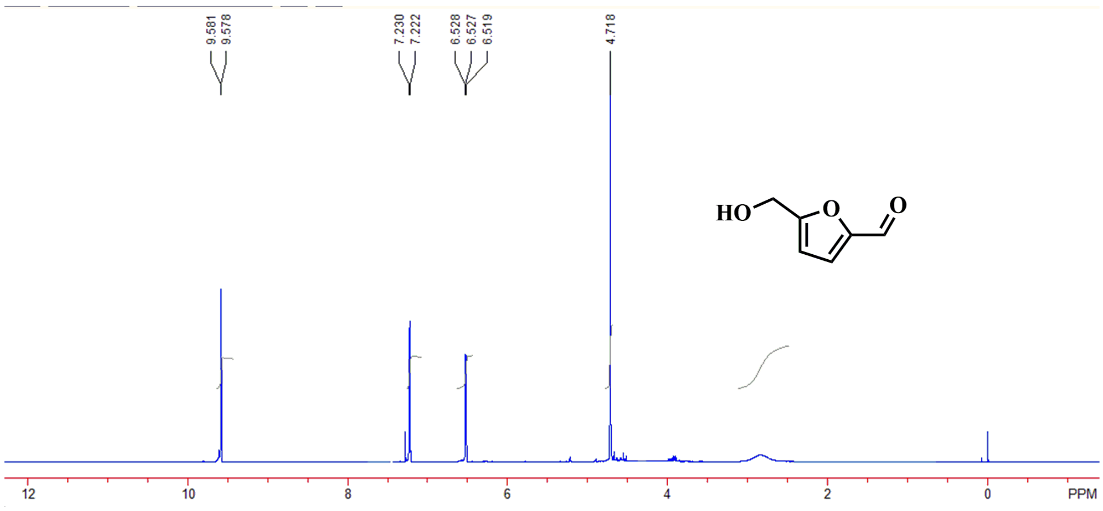


**Supplementary Figure 24 | $^1$H NMR spectrum of the resulting HMF in the dehydration of fructose to HMF, after evaporation of THF.** Reaction conditions: fructose (2.0 g), PSS-30IL-$SO_3$H (2.0 mol%), 120 °C, 10 min, and THF (40 mL) for 10 min. This spectrum confirms that the HMF obtained from this work has high purity.

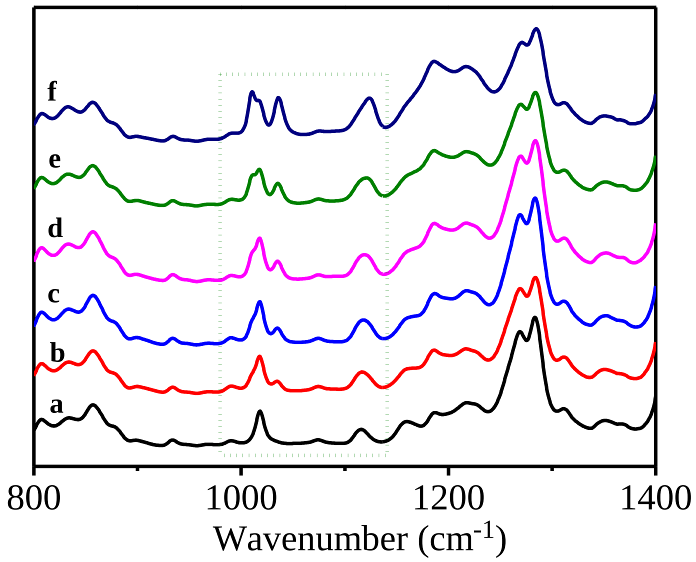


**Supplementary Figure 25 | IR spectra of PSS-xNMP-$SO_3H$.** (a) ∝, (b) 30, (c) 20, (d) 15, (e) 10, and (f) 5, respectively, where x stands for the mole ratio of NMP moieties to sodium *p*-styrene sulfonate. The intensities of bands associated with S=O and C-S increase along with the mole ratio of ionic moiety to $SO_3H$ group.

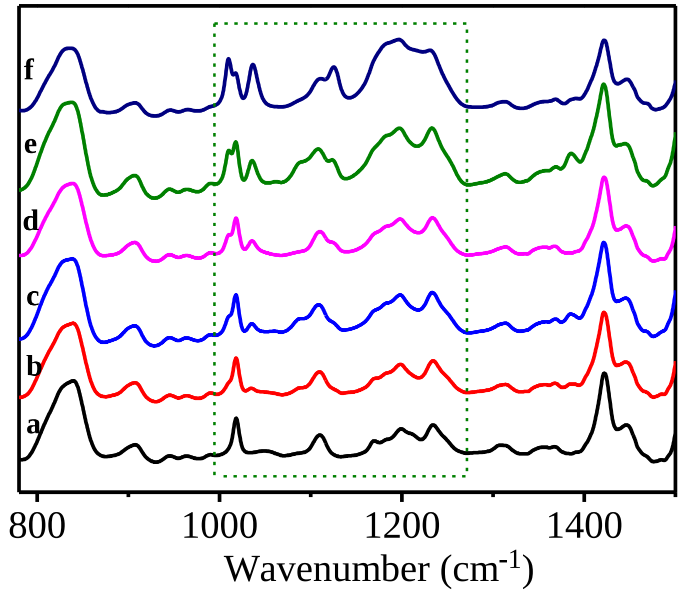


**Supplementary Figure 26 | IR spectra of PSS-xDMSO-$SO_3H$.** (a) ∝, (b) 30, (c) 20, (d) 15, (e) 10, and (e) 5, respectively, where *x* stands for the mole ratios of DMSO moieties to sodium *p*-styrene sulfonate. The intensities of bands associated with S=O and C-S increase along with the mole ratio of ionic moiety to $SO_3H$ group.

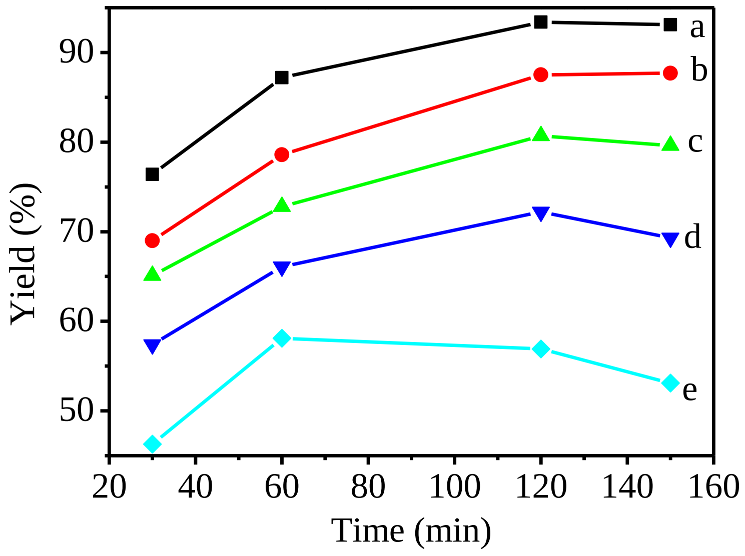


**Supplementary Figure 27 | Plots of HMF yields versus time over various catalysts.** (a) PSS-30NMP-$SO_3H$, (b) PSS-20NMP-$SO_3H$, (c) PSS-15NMP-$SO_3H$, (d) PSS-10NMP-$SO_3H$, and (e) PSS-5NMP-$SO_3H$. Reaction conditions: fructose (100 mg), catalyst (1.0 mol%), THF (5.0 mL), and 120 °C for 2 h.

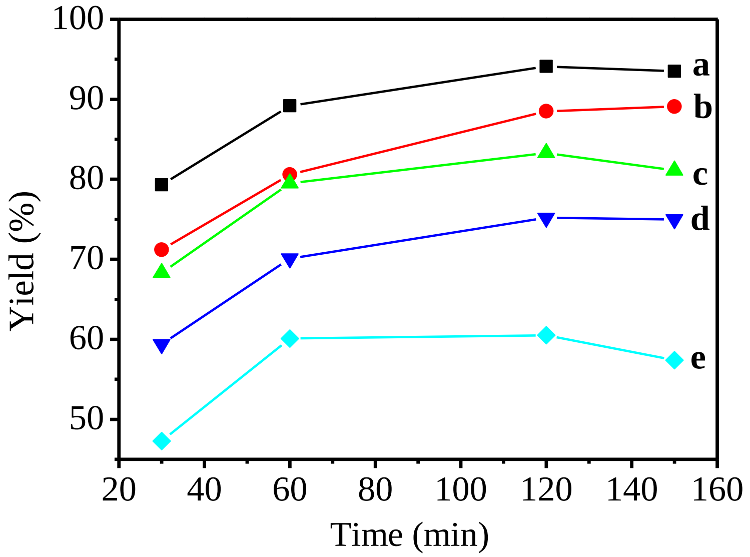


**Supplementary Figure 28 | Plots of HMF yields versus time over various catalysts.** (a) PSS-30NMP-$SO_3H$, (b) PSS-20NMP-$SO_3H$, (c) PSS-15NMP-$SO_3H$, (d) PSS-10NMP-$SO_3H$, and (e) PSS-5NMP-$SO_3H$. Reaction conditions: fructose (100 mg), catalyst (1.0 mol%), THF (5.0 mL), and 120 °C for 2 h.

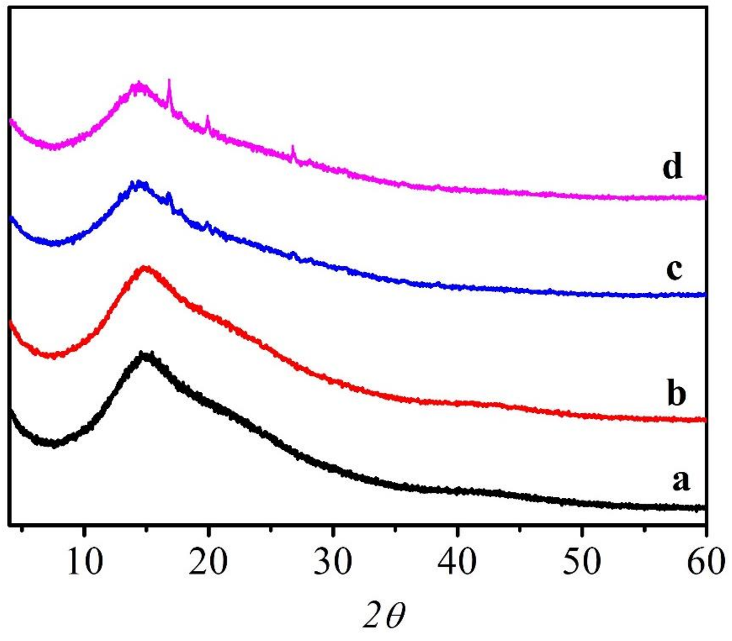


**Supplementary Figure 29 | XRD patterns of finely grinded fructose (10 mg) and polymers (100 mg).** (a) PSS-NMP, (b) PSS-30NMP-$SO_3Na$, (c) PSS-10NMP-$SO_3Na$, and (d) PSS-5NMP-$SO_3Na$, respectively.

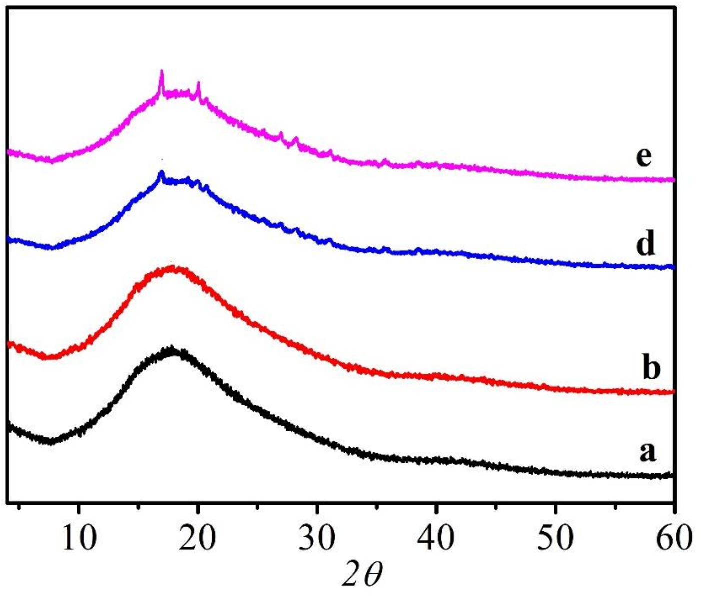


**Supplementary Figure 30 | XRD patterns of finely grinded fructose (10 mg) and polymers (100 mg).** (a) PSS-DMSO, (b) PSS-30DMSO-$SO_3Na$, (c) PSS-10DMSO-$SO_3Na$, and (d) PSS-5DMSO-$SO_3Na$, respectively.

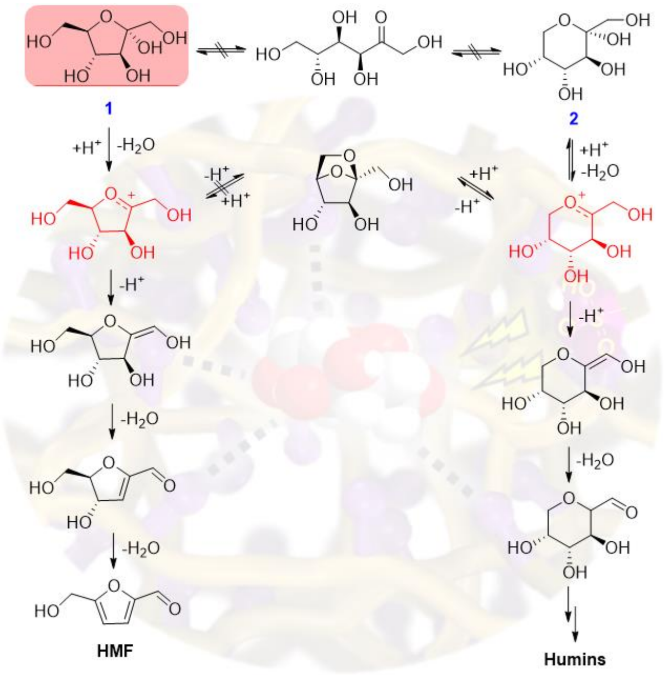


**Supplementary Figure 31 | A representation of the tentatively proposed catalytic route for the dehydration of fructose to HMF catalyzed by PSSs-$SO_3H$.** The solvent moieties in PSSs act as a polar aprotic solvent, functioning as a proton acceptor and forming a hydrogen-bonding network with the hydroxyl groups on fructose, increasing the stability of the furanose tautomers **1** over **6**, and resulting in increased HMF selectivity as well as reducing humin formation.

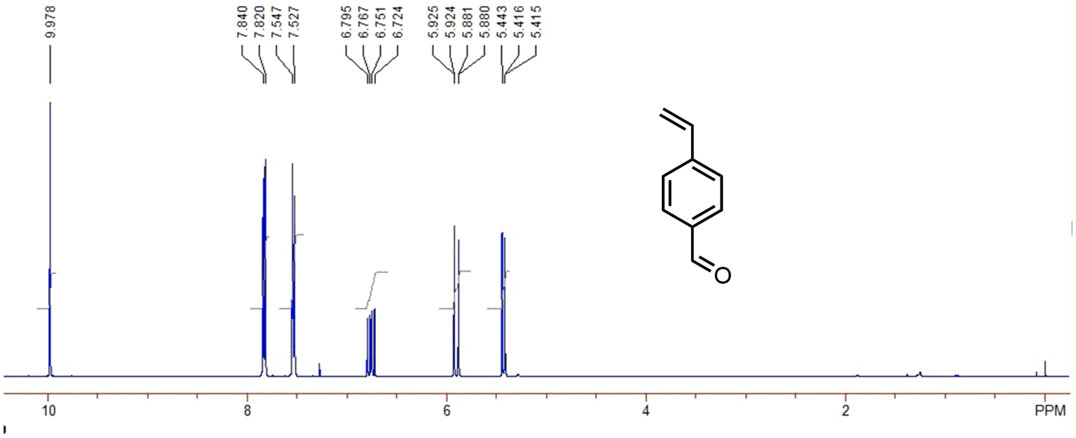
9.978
7.840 7.820 7.547 7.527
6.795 6.767 6.751 6.724
5.925 5.924 5.881 5.880 5.443 5.416 5.415
O
10 8 6 4 2 PPM

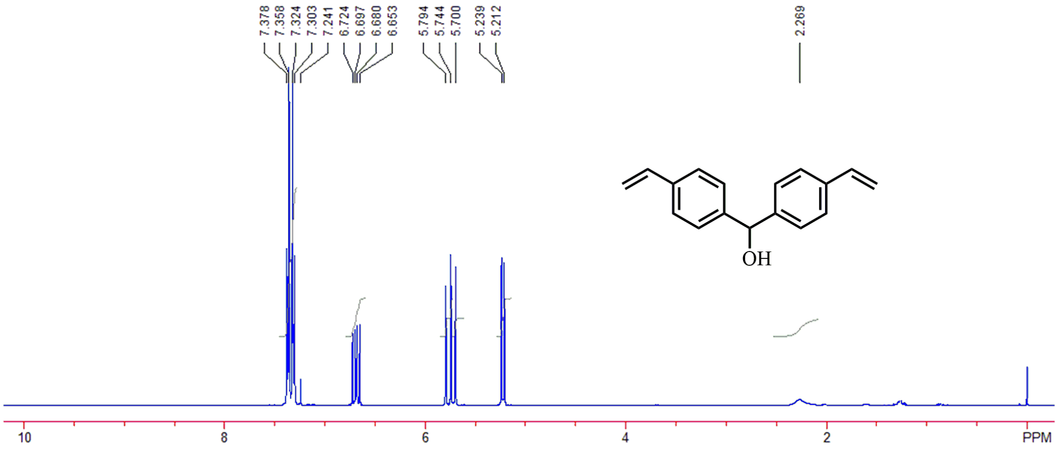
7.378 7.358 7.324 7.303 7.241
6.724 6.697 6.680 6.653
5.794 5.744 5.700
5.239 5.212
2.269
OH
10 8 6 4 2 PPM

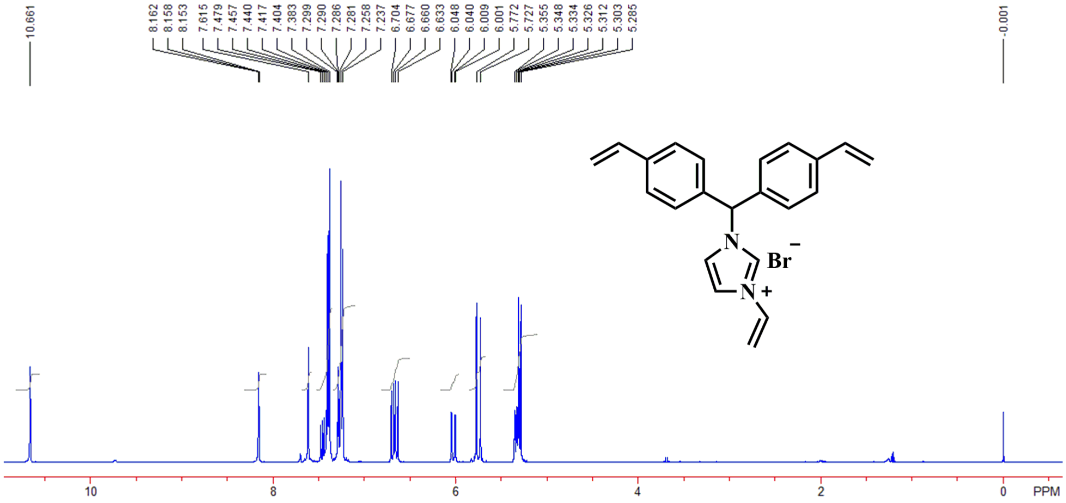
10.661
8.162 8.158 8.153 7.615 7.479 7.457 7.440 7.417 7.404 7.383 7.299 7.290 7.286 7.281 7.258 7.237
6.704 6.677 6.660 6.633
6.048 6.040 6.009 6.001 5.772 5.727 5.355 5.348 5.334 5.326 5.312 5.303 5.285
-0.001
N
Br−
N+
10 8 6 4 2 0 PPM

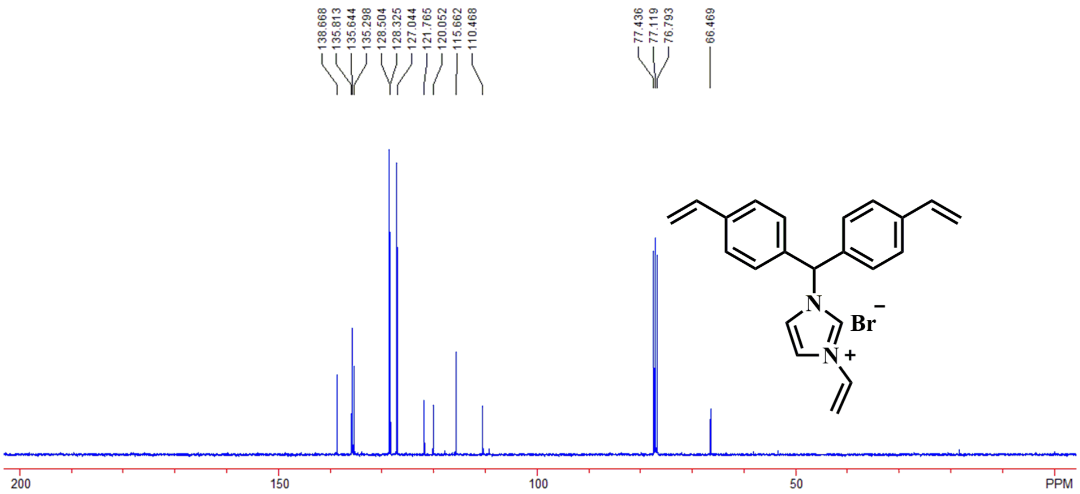

138.668
135.813
135.644
135.298
128.504
128.325
127.044
121.765
120.052
115.662
110.468
77.436
77.119
76.793
66.469
Br
N
N
200
150
100
50
PPM


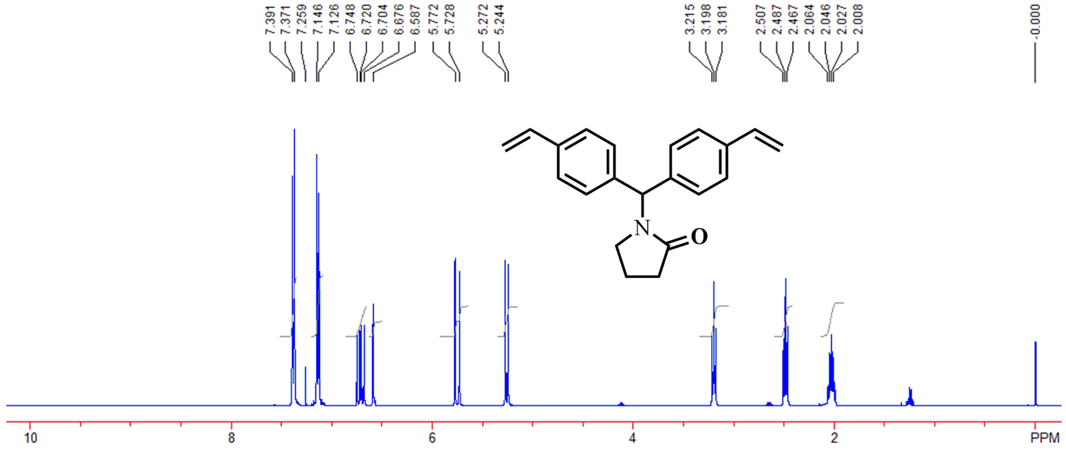

7.391
7.371
7.259
7.146
7.126
6.748
6.720
6.704
6.676
6.587
5.772
5.728
5.272
5.244
3.215
3.198
3.181
2.507
2.487
2.467
2.064
2.046
2.027
2.008
-0.000
N
O
10
8
6
4
2
0
PPM


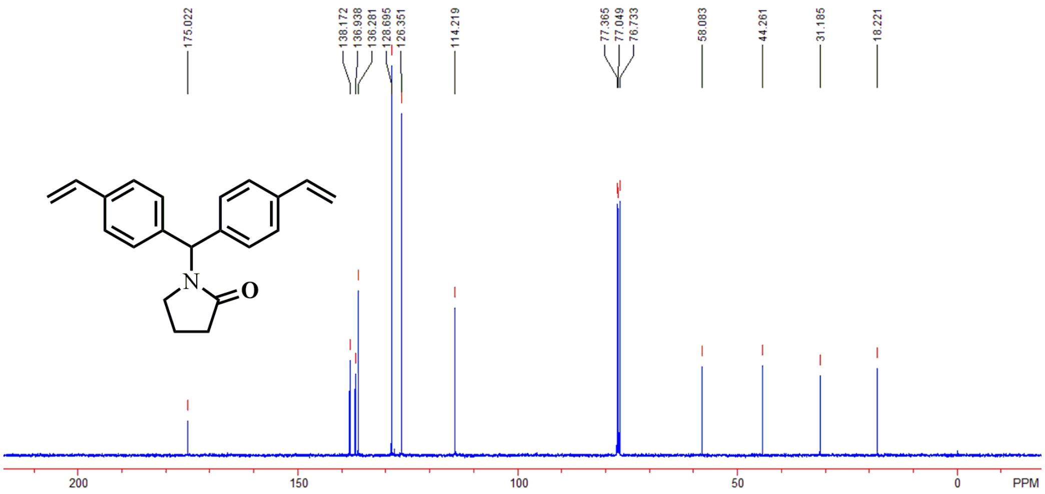

175.022
138.172
136.938
136.281
128.695
126.351
114.219
77.365
77.049
76.733
58.083
44.261
31.185
18.221
N
O
200
150
100
50
0
PPM

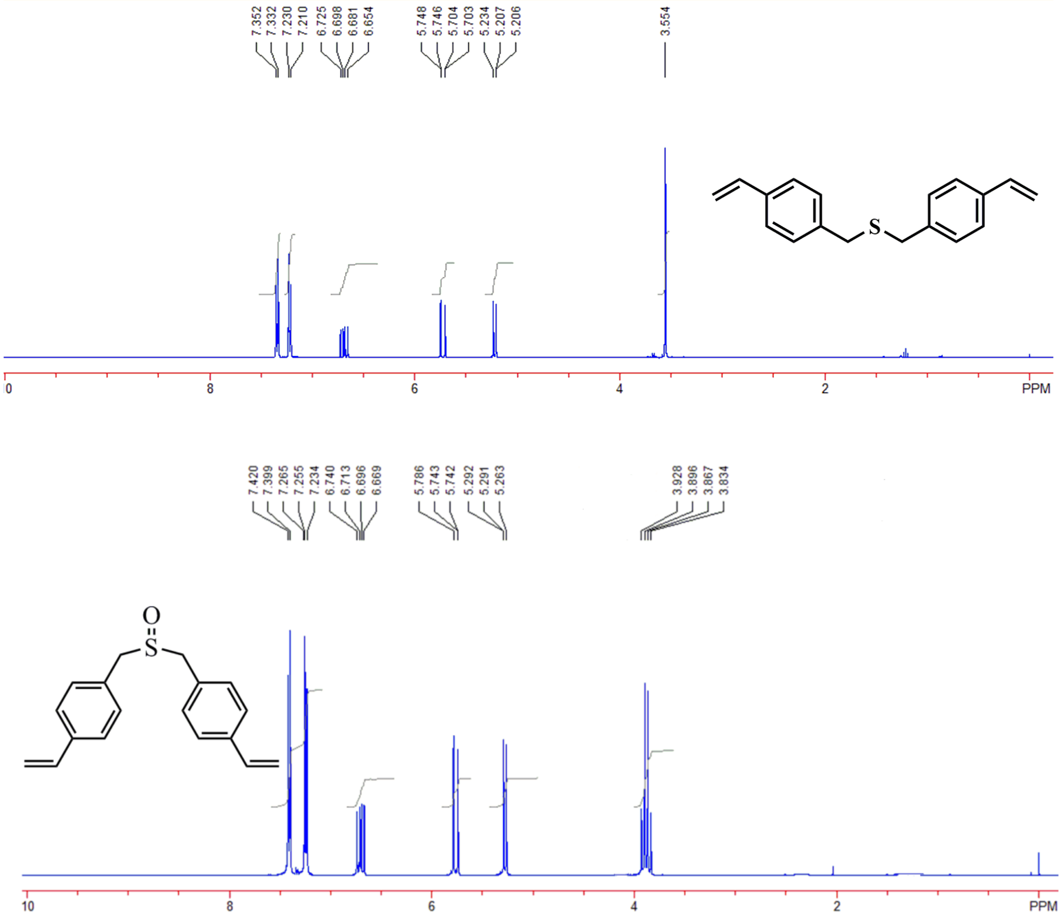

**Supplementary Figure 32 | NMR spectra of various compounds.**

## Supplementary Tables

**Supplementary Table 1 | Textural parameters of the PSSs before and after being treated in boiling water for one week (in parenthesis).**

| PSS | BET surface area ($m^2\ g^{-1}$) | Pore volume ($cm^3\ g^{-1}$) |
|---|---|---|
| PSS-IL | 460 (437) | 0.60 (0.59) |
| PSS-NMP | 596 (592) | 0.53 (0.53) |
| PSS-DMSO | 233 (236) | 0.54 (0.54) |

**Supplementary Table 2 | The acid content of PSS-xIL-$SO_3H$ evaluated by various techniques.**

| x | Theoretical values | Carbon-sulfur analysis | Acid-base titration |
|---|---|---|---|
| 30 | 0.093 | 0.090 | 0.089 |
| 20 | 0.139 | 0.141 | 0.133 |
| 15 | 0.184 | 0.176 | 0.183 |
| 10 | 0.295 | 0.287 | 0.302 |
| 5 | 0.517 | 0.507 | 0.506 |

[a] the unit of acid content is mmol $g^{-1}$

**Supplementary Table 3 | The textural parameters of PSS-xIL-$SO_3H$.**

| x | BET Surface Area ($m^2\ g^{-1}$) | Pore Volume ($cm^3\ g^{-1}$) |
|---|---|---|
| $\propto$ | 460 | 0.60 |
| 30 | 500 | 0.81 |
| 20 | 487 | 0.77 |
| 15 | 380 | 0.64 |
| 10 | 365 | 0.65 |
| 5 | 367 | 0.60 |

**Supplementary Table 4 | The effect of additives on the catalytic performance in dehydration of fructose to HMF over various catalysts. [a]**

| Entry | Catalyst | Additive | Yield (%) |
|---|---|---|---|
| 1 | -- | -- | <1.0 |
| 2 | -- | PSS-IL | <1.0 |
| 3 | -- | PSS-NMP | <1.0 |
| 4 | -- | PSS-DMSO | <1.0 |
| 5 | TsOH | PSS-IL | 64.5 |
| 6 | TsOH | PSS-NMP | 41.4 |
| 7 | TsOH | PSS-DMSO | 43.1 |
| 8 | Amberlyst-15 | PSS-IL | 14.2 |
| 9 | Amberlyst-15 | PSS-NMP | 13.3 |
| 10 | Amberlyst-15 | PSS-DMSO | 13.7 |

[a] Reaction conditions: fructose (100 mg), THF (5.0 mL), catalyst (1.0 mol%), additive (100 mg), and reaction at 120 °C for 2 h.

**Supplementary Table 5 | Catalytic data in dehydration of fructose to HMF over various catalysts.** [a]

| Entry | Catalyst | Solvent | Time (min) [c] | Conv.(%) [c] | Select.(%) [c] | Yield (%) [c] |
|---|---|---|---|---|---|---|
| 1 | PSS-20IL-$SO_3H$ | THF | 60 | >99.5 | 94.6 | 94.6 |
| 2 | PSS-15IL-$SO_3H$ | THF | 60 | >99.5 | 93.1 | 93.1 |
| 3 | PSS-10IL-$SO_3H$ | THF | 60 | 92.5 | 85.5 | 79.1 |
| 4 | PSS-30NMP-$SO_3H$ | THF | 120 | >99.5 | 93.4 | 93.4 |
| 5 | PSS-30DMSO-$SO_3H$ | THF | 120 | >99.5 | 94.1 | 94.1 |
| 6 | Amberlyst-15 | EMIMBr | 120 (10) | >99.5 (91.6) | 39.1 (51.5) | 39.1 (47.2) |
| 7 | Amberlyst-15 | NMP | 120 (10) | 96.6 (7.7) | 74.1 (92.2) | 71.6 (7.1) |
| 8 | Amberlyst-15 | DMSO | 120 (10) | >99.5 (11.8) | 87.4 (93.2) | 87.4 (11.0) |
| 9 | PDVB-$SO_3H$ [b] | EMIMBr | 180 | >99.5 | 95.2 | 95.2 |
| 10 | PDVB-$SO_3H$ | NMP | 180 | >99.5 | 96.3 | 96.3 |
| 11 | PDVB-$SO_3H$ | DMSO | 180 | >99.5 | 98.2 | 98.2 |
| 12 | PDVB-$SO_3H$ | THF | 180 | 65.3 | 37.8 | 24.7 |

[a] Reaction conditions: fructose (100 mg), solvent (5.0 mL), or EMIMBr (1-ethyl-3-methylimidazolidine, bromide salt, 2.0 g), and 120 °C. [b] PDVB-$SO_3H$ was synthesized from the copolymerization of divinylbenzene with sodium *p*-styrene sulfonate, at a mole ratio of 30 to 1, followed by ion-exchange with HCl, exhibiting a BET surface area of 711 $m^2$ $g^{-1}$. [c] The values in parentheses refer to the time used, as well as the conversion of fructose and selectivity and yield of HMF at that point.

We ascribe the disparity in selectivity of HMF catalyzed by PSS-30IL-$SO_3H$ or Amberlyst-15 with corresponding solvent to their difference in acid strength. The $^{31}P$ MAS NMR spectra of trimethylphosphine oxide (TMPO) after interaction with PSS-30IL-$SO_3H$ and Amberlyst-15 samples shows a singlet peak at 64.8 and 82.5 ppm, respectively, indicating that the TMPO molecules exhibit higher interaction strength with Brønsted acid sites in Amberlyst-15 than those in PSS-30IL-$SO_3H$ and PSS-5IL-$SO_3H$ (see details in Supplementary Fig. 21). It is well documented that large amounts of strong acid sites may lead to side reactions and thus reduce the yield of HMF[1-3]. To rationalize our assumption, a porous polymer-based catalyst (PDVB-$SO_3H$) with similar acid strength to that of PSS-30IL-$SO_3H$ was used for comparison. Significant improvements were observed when the reactions were operated in EMIMBr, DMSO, and NMP in the presence of PDVB-$SO_3H$ compared to Amberlyst-15. However, when the reaction was performed use THF as the reaction medium, a very low HMF selectivity was obtained (entry 12), confirming the importance of the desired solvation environment around the active sites to achieve high performance.

**Supplementary Table 6 | Performance of representative catalysts in dehydration of fructose to HMF in the literature.**

| Catalyst | Solvent | Conv. (%) | Select. (%) | Yield (%) | Ref. |
|---|---|---|---|---|---|
| PSS-30IL-$SO_3H$ | THF | >99.5 | 98.8 | 98.8 | this work |
| PSS-30NMP-$SO_3H$ | THF | >99.5 | 93.4 | 93.4 | this work |
| PSS-30DMSO-$SO_3H$ | THF | >99.5 | 94.1 | 94.1 | this work |
| PVP-*p*$SO_3H$-SBA-15 | THF&$H_2O$ | 50 | 78 | 39 | 4 |
| $H_2SO_4$ | [BMIM][Cl] | 90-99 | 80-90 | 72-89 | 5 |
| NHC/Cr | [BMIM][Cl] | -- | -- | 65-96 | 6 |
| PSSH-grafted silica particles | $H_2O$ | 91 | 34 | 31 | 7 |
| trifluoromethanesulfonic acid | [MIM][Cl] | 96 | 91.6 | 88 | 8 |
| Sn-Beta, HCl | Biphasic ($H_2O$/THF/NaCl) | 79 | 72 | 56 | 9 |
| TESAS-SBA-15 | 7:3 MIBK:2-butanol | 84 | 71 | 60 | 10 |
| $[NMP]^+[HSO_4]^-$ | DMSO | 98.6 | 70.4 | 69.4 | 11 |
| Nafion(15)/MCF | DMSO | 94 | 95.0 | 89.3 | 12 |
| HCl | $ZnCl_2$ aqueous solution | 97.3 | 54.8 | 53.3 | 13 |
| Glu-TsOH | DMSO(NMP) | 99.9(97.9) | 91.2(87.6) | 91.1(85.8) | 14 |
| Amberlyst-15 | [BMIM][Cl] | 99.4 | 83.7 | 83.2 | 15 |
| Si-3-IL-$HSO_4$ | DMSO | 99.9 | 63.3 | 63.3 | 16 |
| Titanate nanotube | $H_2O$ | -- | -- | ~16 | 17 |
| [EMIM]Cl | -- | >99.5 | ~73 | ~73 | 18 |
| HCl | 7:3(8:2 Water:DMSO):PVP<br>7:3 MIBK:2-butanol | 89 | 85 | 75.7 | 19 |
| P-$SO_3H$-154 | DMSO | -- | -- | 99 | 20 |

**Supplementary Table 7 |Textural parameters for PSS-xIL-DVB synthesized from various mole ratios of V-IL to divinylbenzene (DVB).**

| x [a] | BET Surface Area ($m^2$ $g^{-1}$) | Pore Volume ($cm^3$ $g^{-1}$) |
|---|---|---|
| 30 | 492 | 0.47 |
| 20 | 412 | 0.47 |
| 15 | 413 | 0.50 |
| 10 | 410 | 0.61 |
| 5 | 439 | 0.64 |
| 2 | 498 | 0.85 |
| 1 | 506 | 0.98 |

[a] x stands for mole ratio of V-IL to DVB.

**Supplementary Table 8 | Effects of reaction temperature and solvent on the yields of HMF. [a]**

| Entry | Temperature (°C) | Solvent | Time (min) | Yield (%) |
|---|---|---|---|---|
| 1 | 120 | THF | 10 | 98.8 |
| 2 | 100 | THF | 60 | 97.8 |
| 3 | 80 | THF | 150 | 96.1 |
| 4 | 60 | THF | 540 | 95.2 |
| 5 | 40 | THF | 1440 | 71.0 |
| 6 [b] | 120 | $H_2O$ | 300 (10) | 7.2 (<1.0) |
| 7 [b] | 120 | THF/$H_2O$ = 10/1 | 240 (10) | 76.7 (10.3) |
| 8 [b] | 120 | THF/$H_2O$ = 20/1 | 120 (10) | 89.4 (19.8) |

[a] Reaction conditions: fructose (100 mg), THF (5.0 mL), PSS-30IL-$SO_3H$ (1.0 mol% based on $H^+$ species). [b] The values in parentheses refer to the time used, as well as the selectivity and yield of HMF at this point.

**Supplementary Table 9 | Recycling tests of PSS-30IL-$SO_3H$ in dehydration of fructose to HMF.**

| Run | Yield (%) |
|---|---|
| 0 | 98.7 |
| 1 | 98.5 |
| 2 | 98.8 |
| 3 | 98.1 |
| 4 | 98.1 |
| 5 | 97.8 |

Reaction conditions: fructose (100 mg), catalyst (1.0 mol%), THF (5.0 mL), 120 °C, and 10 min.

**Supplementary Table 10 | Recycling tests of PSS-30IL-$SO_3H$ in dehydration of fructose to HMF.**

| Run | Conversion (%) | Yield (%) |
|---|---|---|
| 0 | 61.2 | 59.6 |
| 1 | 61.7 | 60.2 |
| 2 | 60.1 | 58.8 |
| 3 | 59.1 | 57.1 |
| 4 | 60.3 | 59.0 |
| 5 | 58.3 | 57.8 |

Reaction conditions: fructose (200 mg), catalyst (1.0 mol%), THF (5.0 mL), 120 °C, and 5 min.

**Supplementary Table 11 | Textural parameters of PSS-xNMP-$SO_3H$ synthesized from various mole ratios of V-NMP to sodium *p*-styrene sulfonate.**

| x [a] | BET Surface Area ($m^2$ $g^{-1}$) | Pore Volume ($cm^3$ $g^{-1}$) |
|---|---|---|
| ∝ | 596 | 0.53 |
| 30 | 543 | 0.46 |
| 20 | 536 | 0.38 |
| 15 | 498 | 0.29 |
| 10 | 438 | 0.28 |
| 5 | 378 | 0.23 |

[a] x stands for mole ratio of V-NMP to sodium *p*-styrene sulfonate.

**Supplementary Table 12 | Textural parameters for PSS-xDMSO-$SO_3H$ synthesized from various mole ratios of V-DMSO to sodium *p*-styrene sulfonate.**

| x [a] | BET Surface Area ($m^2$ $g^{-1}$) | Pore Volume ($cm^3$ $g^{-1}$) |
|---|---|---|
| ∝ | 233 | 0.54 |
| 30 | 222 | 0.54 |
| 20 | 233 | 0.49 |
| 15 | 170 | 0.43 |
| 10 | 151 | 0.25 |
| 5 | 141 | 0.19 |

[a] x stands for mole ratio of V-DMSO to sodium *p*-styrene sulfonate.

**Supplementary Table 13 | Textural parameters for PSS-xIL-IM synthesized from various mole ratios of V-IL to 1-vinylimidazole.**

| x [a] | BET Surface Area ($m^2$ $g^{-1}$) | Pore Volume ($cm^3$ $g^{-1}$) |
|---|---|---|
| ∝ | 460 | 0.60 |
| 30 | 443 | 0.58 |
| 20 | 410 | 0.53 |
| 15 | 404 | 0.52 |
| 10 | 351 | 0.46 |
| 5 | 317 | 0.44 |

[a] x stands for mole ratio of V-IL to 1-vinylimidazole.

**Supplementary Table 14 | Catalytic performance of the cascade base-catalyzed isomerization of glucose to fructose and acid-catalyzed dehydration of fructose to HMF over various catalysts. [a]**

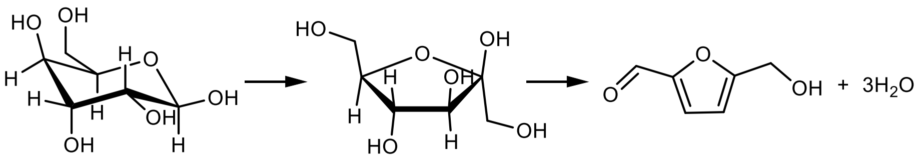


| Catalyst | Conversion (%) | HMF yield (%) |
|---|---|---|
| PSS-30IL-$SO_3H$ + PSS-30IL-IM | >99.5 | 84.3 |
| PSS-20IL-$SO_3H$ + PSS-20IL-IM | 97.6 | 78.2 |
| PSS-15IL-$SO_3H$ + PSS-15IL-IM | 86.2 | 69.1 |
| PSS-10IL-$SO_3H$ + PSS-10IL-IM | 60.3 | 43.3 |
| PSS-5IL-$SO_3H$ + PSS-5IL-IM | 40.2 | 19.5 |
| PSS-5IL-$SO_3H$ + PSS-30IL-IM | 46.4 | 23.6 |
| PDVB-$SO_3H$ + PDVB-IM | 28.1 | 6.2 |

Reaction conditions: fructose (100 mg, 0.56 mmol), acid catalyst (based on the amount of $SO_3H$ 1.0 mol%), base catalyst (based on the amount of imidazole 1.0 mol%), 120 °C, 3 h, THF (5.0 mL). The BET surface area of PDVB-$SO_3H$ and PDVB-IM were calculated to be 711 and 594 $m^2$ $g^{-1}$, respectively.

# Supplementary Methods

## Material synthesis

### *Synthesis of 1-(bis(4-vinylphenyl)methyl)-3-vinylimidazolidine, bromide salt*

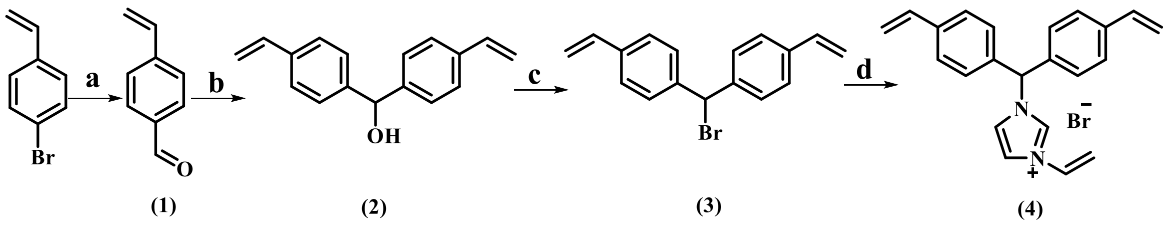

Reagents: (a) Mg, DMF; (b) 4-bromostyrene, Mg; (c) $PBr_3$; (d) 1-vinylimidazole

*4-vinylbenzaldehyde* **(1)**. To a solution of (4-vinylphenyl)magnesium bromide, which was prepared by treatment of 4-bromostyrene (100 mmol) and Mg powder (120 mmol) in THF, DMF (150 mmol) was added dropwise at 0 °C under $N_2$. After being stirred at room temperature overnight, the reaction was quenched by addition of saturated $NH_4Cl$ aqueous solution. The residue was extracted with ethyl acetate, washed with brine, dried over $Na_2SO_4$, and evaporated under reduced pressure, giving the crude compound which was purified by flash chromatography on silica gel (hexane:EtOAc = 10:1) as eluent to afford the title compound as a yellow oil. Yield: 12.1 g (92%). $^1H$ NMR (400 MHz, $CDCl_3$, 298K, TMS): δ 9.98 (s, 1H), 7.83 (d, 2H, *J*=8.0Hz), 7.54 (d, 2H, *J*=8.0Hz), 6.72-6.80 (m, 1H), 5.88-5.93 (m, 1H), 5.42 (t, 1H, *J*=5.6Hz) ppm.

*bis(4-vinylphenyl)methanol* **(2)**. To a solution of (4-vinylphenyl)magnesium bromide, which was prepared by treatment of 4-bromostyrene (60 mmol) and Mg powder (72 mmol) in THF, **1** (50 mmol) was added dropwise at 0 °C under $N_2$. After being stirred at room temperature overnight, the reaction was quenched by addition of saturated $NH_4Cl$ aqueous solution. The residue was extracted with ethyl acetate, washed with brine, dried over $Na_2SO_4$, and evaporated under

reduced pressure, giving the crude compound which was purified by flash chromatography on silica gel (hexane:EtOAc = 10:1) as eluent to afford the title compound as a white solid. Yield: 11.2 g (95%). $^1H$ NMR (400 MHz, $CDCl_3$, 298K, TMS): δ 7.24-7.34 (m, 8H), 6.65-6.72 (m, 2H), 5.77 (d, 2H, *J*=20 Hz), 5.70 (s, 1H), 5.23 (d, 2H, *J*=10.8 Hz), 2.27 (s, 1H) ppm.

*4,4'-(bromomethylene)bis(vinylbenzene)* **(3)**. $PBr_3$ (3.4 g) was added slowly to the solution of **2** (2.0 g) in 80 mL of ether at 0 °C. The resulting suspension was stirred at room temperature for 1 h, and then another portion of $PBr_3$ (3.4 g) was introduced. After being stirred for another 1 h, the reaction was quenched by addition of $H_2O$ (100 mL). The residue was extracted with ether, washed with saturated $NaHCO_3$, and dried over $Na_2SO_4$. Evaporation of the solvents afforded **3** as a white solid, which was used without further purification[21].

*1-(bis(4-vinylphenyl)methyl)-3-vinylimidazolidine, bromide salt* **(4)**. **3** (2.0 g) and 1-vinylimidazole (0.7 g) was dissolved in acetone (20 mL), stirring at 60 °C under $N_2$ atmosphere for 12 h. After the reaction, the mixture was cooled to room temperature, followed by filtration, washing with diethyl ether, and drying under vacuum at room temperature. The product was obtained as a white solid in quantitative

yield. $^{1}H$ NMR (400 MHz, $CDCl_3$, 298K, TMS): δ 10.67 (s, 1H), 8.15 (t, 1H, *J*=1.8Hz), 7.62 (s, 1H), 7.42-7.48 (m, 1H), 7.39 (d, 4H, *J*=8.4 Hz), 7.28-7.30 (m, 1H), 7.25 (d, 4H, *J*=8.4 Hz), 6.63-6.70 (m, 2H), 6.00-6.05 (m, 1H), 5.75 (d, 2H, *J*=18 Hz), 5.33-5.36 (m, 1H), 5.30 (d, 2H, *J*=10.8 Hz) ppm. $^{13}C$ NMR (100 MHz, $CDCl_3$) δ 66.47, 110.47, 115.66, 120.05, 121.77, 127.04, 128.32, 128.50, 135.30, 135.64, 135.81, 138.67 ppm.

***Synthesis of 1-(bis(4-vinylphenyl)methyl)pyrrolidin-2-one (5)***

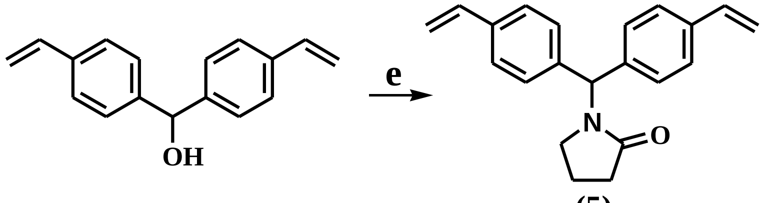


**(5)**

Reagents: (e) 2-pyrrolidinone, *p*-toluenesulfonic acid

*1-(bis(4-vinylphenyl)methyl)pyrrolidin-2-one* **(5)**. To a suspension of 2-pyrrolidinone (2.88 g, 33.8 mmol) in cyclohexane (300 mL), *p*-toluenesulfonic acid monohydrate (3.38 g, 16.9 mmol) and **2** (4.0 g, 16.9 mmol) were introduced. After being refluxed for 24 h, the residue was extracted with ethyl acetate, washed with brine, dried over $Na_2SO_4$, and evaporated under reduced pressure, giving the crude compound which was purified by flash chromatography on silica gel (hexane:EtOAc = 2:1) as eluent to afford the title compound as a white solid. Yield: 3.29 g (64%). $^{1}H$ NMR (400 MHz, $CDCl_3$, 298K, TMS): δ 7.38 (d, 4H, *J*=8.0 Hz), 7.14 (d, 4H, *J*=8.0 Hz), 6.68-6.75 (m, 2H), 6.59 (s, 1H), 5.75 (d, 2H, *J*=17.6 Hz), 5.26 (d, 2H, *J*=11.2 Hz), 3.2 (t, 2H, 6.8Hz), 2.49 (t, 2H, *J*=8.0 Hz), 1.99-2.06 (m, 2H) ppm. $^{13}C$ NMR (100 MHz, $CDCl_3$) δ 18.22, 31.19, 44.26, 58.08, 114.22, 126.35, 128.70, 136.28, 136.94, 138.17, 175.02 ppm.

***Synthesis of bis(4-vinylbenzyl)sulfane (6)***

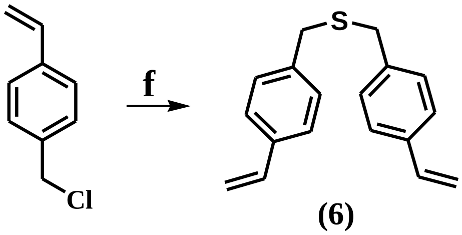


**(6)**

Reagents: (f) $Na_2S{\cdot}9H_2O$

To a solution of 4-vinylbenzyl chloride (15.2 g, 100 mmol) in ethanol (60 mL) at room temperature, $Na_2S{\cdot}9H_2O$ aqueous solution (12.02 g dissolved in 20 mL of $H_2O$) was added in dropwise. After being refluxed for 0.5 h, the resulting mixture was poured into ice water, filtrated, and purified by flash chromatography on silica gel (hexane:EtOAc = 10:1) as eluent to afford the title compound as a white solid. Yield: 12.3 g (92.8%).$^{1}H$ NMR (400 MHz, $CDCl_3$, 298K, TMS): δ 7.34 (d, 4H, *J*=8.0 Hz), 7.22 (d, 4H, *J*=8.0 Hz), 6.65-6.73 (m, 2H), 5.70-5.75 (m, 2H), 5.22 (t, 2H, *J*=5.6 Hz), 3.55 (s, 4H) ppm.

***Synthesis of PSS-IL***

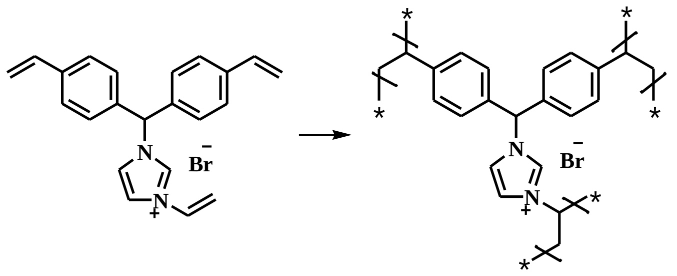


1.0 g of **4** was dissolved in 10 mL of DMF, followed by addition of 50 mg of AIBN. The mixture was transferred into an autoclave at 100 ºC for 24 h. The title polymer was obtained as a light yellow solid after being washed with ethanol and evaporated under a vacuum (*ca.* 1.0 g, quantitative yield).

***Synthesis of PSS-NMP***

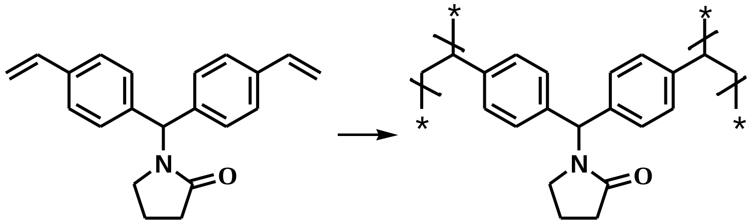


1.0 g of **5** was dissolved in 10 mL of DMF, followed by the addition of 50 mg of AIBN. The mixture was transferred into an autoclave at 100 ºC for 24 h. The title polymer was obtained as a white solid after being washed with ethanol and evaporated under a vacuum (*ca.* 1.0 g, quantitative yield).

***Synthesis of PSS-DMSO***

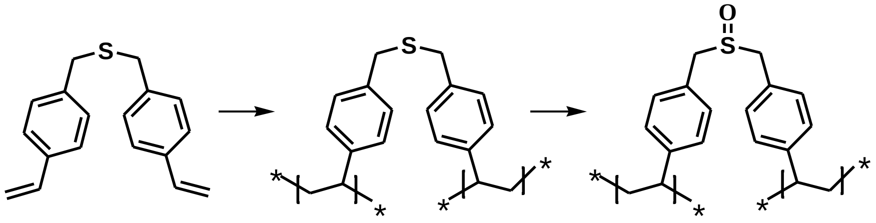


PSS-DMSO was synthesized in two steps. Firstly, **6** was polymerized under solvothermal conditions, as described above. Typically, 1.0 g of **6** was dissolved in 10 mL of DMF, followed by the addition of 50 mg of AIBN. The mixture was transferred into an autoclave at 100 ºC for 24 h. The title polymer was obtained as a white solid after being washed with ethanol and evaporated under a vacuum (*ca.* 1.0 g, quantitative yield). The obtained polymer was oxidized by $H_2O_2$ in the presence of $POCl_3$[22].

***Synthesis of PSS-xIL-SO$_3$H (x stands for the mole ratio of ionic moiety to sodium p-styrene sulfonate)***

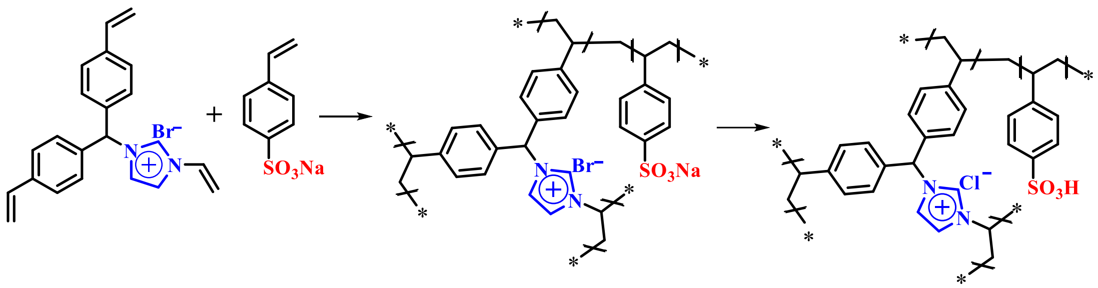

**PSS-xIL-SO$_3$Na** **PSS-xIL-SO$_3$H**

As a typical run, 1.0 g of **4** and 17 mg of sodium *p*-styrene sulfonate were dissolved in 10 mL of DMF, followed by addition of 50 mg of AIBN. The mixture was transferred into an autoclave at 100 °C for 24 h. After being washed with ethanol and dried under a vacuum, the yielded polymer was ion-exchanged using 1 M HCl (100 mL) for 6 h and this procedure was repeated for three times. The resulting sample denoted as PSS-30IL-SO$_3$H, was washed with distilled water thoroughly until the pH of the filtrate was about 7 and then dried at 50 °C under vacuum for 12 h. Other catalysts with different mole ratio of ionic monomer to sulfonic acid group were synthesized follow the same procedure, except that more sodium *p*-styrene sulfonate compound was introduced. The sulfonic acid content in the resultant polymers was measured by ion exchange with NaCl (2 M), followed by titration of the free $H^+$ with NaOH.

***Synthesis of PSS-xNMP-SO$_3$H (x stands for the mole ratio of 5 to sodium p-styrene sulfonate)***

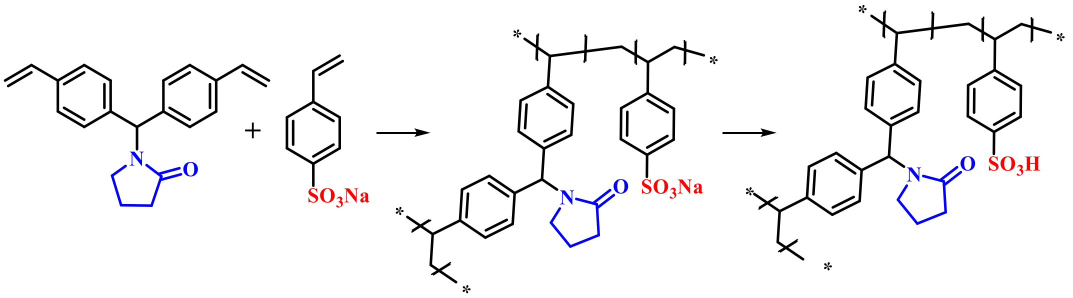

**PSS-xNMP-SO$_3$Na** **PSS-xNMP-SO$_3$H**

As a typical run, 1.0 g of **5** and 23 mg of sodium *p*-styrene sulfonate were dissolved in 10 mL DMF, followed by the addition of 50 mg of AIBN. The mixture was transferred into an autoclave at 100 °C for 24 h. After being washed with ethanol and dried under a vacuum, the yielded polymer was ion-exchanged using 1 M HCl (100 mL) for 6 h and this procedure was repeated for three times. The resulting sample denoted as PSS-30NMP-SO$_3$H was washed with distilled water thoroughly until the pH of the filtrate was about 7 and then dried at 50 °C under vacuum for 12 h. Other catalysts with different mole ratio of NMP contained monomer to sulfonic acid group were synthesized follow the same procedure, except that more sodium *p*-styrene sulfonate compound was introduced.

***Synthesis of PSS-xDMSO-SO$_3$H (x stands for mole ratio of 6 to sodium p-styrene sulfonate).***

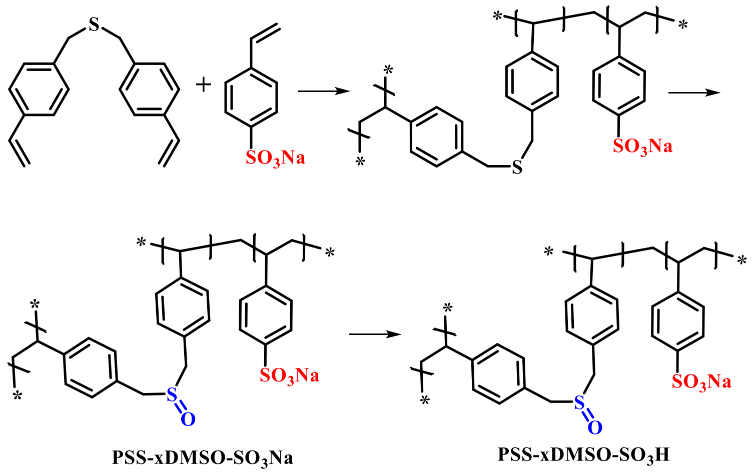


As a typical run, 1.0 g of **6** and 25.8 mg of sodium *p*-styrene sulfonate were dissolved in 10 mL DMF, followed by the addition of 50 mg of AIBN. The mixture was transferred into an autoclave at 100 °C for 24 h. After being washed with ethanol and dried under a vacuum, the yielded polymer was oxidation with $H_2O_2$ in the presence of $POCl_3$[22], followed by ion-exchanged with 1 M HCl as described above.

## ***Catalytic Tests***

**Dehydration of fructose to HMF.** The reactions were carried out in a 50 mL Schlenk tube with a magnetic stirrer in an oil bath. As a typical run, fructose (100 mg), catalyst, and solvent (5 mL) were introduced into the tube. After sealing, the tube was put into a preheated oil bath and stirred at 1200 rpm for a desired time. The yields of HMF were analyzed by gas chromatography (GC) with a flexible quartz capillary column (FFAP) using ethylbenzene as an internal standard. The yield of HMF from conversion of fructose was defined using Supplementary Equation 1.

$$\text{HMF yield (\%)} = \text{moles of HMF produced/moles of starting fructose} * 100 \qquad (1)$$

To determine the conversion of fructose at a certain time, another separate reaction was carried out. After the reaction, the catalyst was separated by centrifugation and washed with water (5 mL x 5). All the liquid was combined and diluted into 50 mL. The concentration of fructose was analyzed by liquid chromatography (LC, Shimadzu LC-10AT) equipped with a RID detector and an Agilent ZORBAX column with a detection limit of 10 ppm. Acetonitrile and water with a volume ratio of 8 to 2 (V/V = 8/2) was used as a fluent with a flow rate of 1.0 mL $min^{-1}$. The temperature of the column was maintained at 30 °C.

For recycling, the catalyst was separated from the reaction system by centrifugation and washed with excessive THF.

## Supplementary References


1. Ma, Z.; Hu, H.; Sun, Z.; Fang, W.; Zhang, J.; Yang, L.; Zhang, Y. & Wang, L. Acidic zeolite L as a highly efficient catalyst for dehydration of fructose to 5-hydroxymethylfurfural in ionic liquid. *ChemSusChem* **10**, 1669-1674 (2017).
2. Neves, P.; Antunes, M. M.; Russo, P. A.; Abrantes, J. P.; Lima, S.; Fernandes, A.; Pillinger, M.; Rocha, S. M.; Ribeiro, M. F.; Valente, A. A. Production of biomass-derived furanic ethers and levulinate esters using heterogeneous acid catalysts. *Green Chem.* **15**, 3367-3376 (2013).
3. Akien, G. R.; Qi, L.; Horváth, I. T. Molecular mapping of acid catalyzed dehydration of fructose. *Chem. Commun.* **48**, 5850-5852 (2012).
4. Alamillo, R.; Crisci, A. J.; Gallo, J. M. R.; Scott, S. L. & Dumesic, J. A. A tailored microenvironment for catalytic biomass conversion in inorganic-organic nanoreactors. *Angew. Chem. Int. Ed.* **52**, 10349-10351 (2013).
5. Galkin, K. I.; Krivodaeva, E. A.; Romashov, L. V.; Zalesskiy, S. S.; Kachala, V. V.; Burykina, J. V. & Ananikov, V. P. Critical influence of 5-hydroxymethylfurfural aging and decomposition on the utility of biomass conversion in organic synthesis. *Angew. Chem. Int. Ed.* **55**, 8338-8342 (2016).
6. Yong, G.; Zhang, Y. & Ying, J. Y. Efficient catalytic system for the selective production of 5-hydroxymethylfurfural from glucose and fructose. *Angew. Chem. Int. Ed.* **120**, 9485-9488 (2008).
7. Tian, C.; Bao, C.; Binder, A.; Zhu, Z.; Hu, B.; Guo, Y.; Zhao, B. & Dai, S. An efficient and reusable "hairy" particle acid catalyst for the synthesis of 5-hydroxymethylfurfural from dehydration of fructose in water. *Chem. Commun.* **49**, 8668-8670 (2013).
8. Shi, C.; Zhao, Y.; Xin, J.; Wang, J.; Lu, X.; Zhang, X. & Zhang, S. Effects of cations and anions of ionic liquids on the production of 5-hydroxymethylfurfural from fructose. *Chem. Commun.* **48**, 4103-4105 (2012).
9. Nikolla, E.; Román-Leshkov, Y.; Moliner, M. & Davis, M. E. "One-pot" synthesis of 5-(hydroxymethyl)furfural from carbohydrates using Tin-Beta zeolite. *ACS Catal.* **1**, 408-410 (2011).
10. Crisci, A. J.; Tucker, M. H.; Lee, M.-Y.; Jang, S. G.; Dumesic, J. A. & Scott, S. L. Acid-functionalized SBA-15-type silica catalysts for carbohydrate dehydration. *ACS Catal.* **1**, 719-728 (2011).
11. Tong, X. & Li, Y. Efficient and selective dehydration of fructose to 5-hydroxymethylfurfural catalyzed by Brønsted-acidic ionic liquids. *ChemSusChem* **3**, 350-355 (2010).
12. Huang, Z.; Pan, W.; Zhou, H.; Qin, F.; Xu, H. & Shen, W. Nafion-resin-modified mesocellular silica foam catalyst for 5-hydroxymethylfurfural production from d-fructose. *ChemSusChem* **6**, 1063-1069 (2013).
13. Deng, T.; Cui, X.; Qi, Y.; Wang, Y.; Hou, X. & Zhu, Y. Conversion of carbohydrates into 5-hydroxymethylfurfural catalyzed by $ZnCl_2$ in water. *Chem. Commun.* **48**, 5494-5496 (2012).
14. Wang, J.; Xu, W.; Ren, J.; Liu, X.; Lu, G. & Wang, Y. Efficient catalytic conversion of fructose into hydroxymethylfurfural by a novel carbon-based solid acid. *Green Chem.* **13**, 2678-2681 (2011).
15. Qi, X.; Watanabe, M.; Aida, T. M. & Smith, Jr., R. L. Efficient process for conversion of fructose to 5-hydroxymethylfurfural with ionic liquids. *Green Chem.* **11**, 1327-1331 (2009).

16. Sidhpuria, K. B.; Daniel-da-Silva, A. L.; Trindade, T. & Coutinho, J. A. P. Supported ionic liquid silica nanoparticles (SILnPs) as an efficient and recyclable heterogeneous catalyst for the dehydration of fructose to 5-hydroxymethylfurfural. *Green Chem.* **13**, 340-349 (2011).

17. Kitano, M.; Nakajima, K.; Kondo, J. N.; Hayashi, S. & Hara, M. Protonated titanate nanotubes as solid acid catalyst. *J. Am. Chem. Soc.* **132**, 6622-6623 (2010).

18. Zhao, H.; Holladay, J. E.; Brown, H. & Zhang, Z. C. Metal chlorides in ionic liquid solvents convert sugars to 5-hydroxymethylfurfural. *Science* **316**, 1597-1600 (2007).

19. Román-Leshkov, Y.; Chheda, J. N. & Dumesic, J. A. Phase modifiers promote efficient production of hydroxymethylfurfural from fructose. *Science* **321**, 1933-1937 (2006).

20. Wang, L.; Wang, H.; Liu, F.; Zheng, A.; Zhang, J.; Sun, Q.; Lewis, J. P.; Zhu, L. F.; Meng, X. & Xiao, F.-S. Selective catalytic production of 5-hydroxymethylfurfural from glucose by adjusting catalyst wettability. *ChemSusChem* **7**, 402-406 (2014).

21. Sellnel, H.; Faber, C.; Rheiner, P. B. & Seebach, D. Immobilization of BINOL by cross-linking copolymerization of styryl derivatives with styrene, and applications in enantioselective Ti and Al Lewis acid mediated additions of $Et_2Zn$ and $Me_3SiCN$ to aldehydes and of diphenyl nitrone to enol ethers. *Chem. Eur. J.* **6**, 3692-3705 (2000).

22. Khodaei, M. M.; Bahrami, K. & Tirandaz, Y. $POCl_3$ as a catalytic activator for $H_2O_2$ activation in selective sulfide oxidation. *J. Sulfur Chem.* **30**, 581-584 (2009).
